# Multiplex Identification of Gram-Positive Bacteria and Resistance Determinants Directly from Positive Blood Culture Broths: Evaluation of an Automated Microarray-Based Nucleic Acid Test

**DOI:** 10.1371/journal.pmed.1001478

**Published:** 2013-07-02

**Authors:** Blake W. Buchan, Christine C. Ginocchio, Ryhana Manii, Robert Cavagnolo, Preeti Pancholi, Lettie Swyers, Richard B. Thomson, Christopher Anderson, Karen Kaul, Nathan A. Ledeboer

**Affiliations:** 1 Department of Pathology, Medical College of Wisconsin, Milwaukee, Wisconsin, United States of America; 2 North Shore-LIJ Health System, New York, New York, United States of America; 3 Hofstra North Shore-LIJ School of Medicine, Hempstead, New York, United States of America; 4 med fusion LLC, Lewisville, Texas, United States of America; 5 Department of Pathology, The Ohio State University Medical Center, Columbus, Ohio, United States of America; 6 Department of Pathology; NorthShore University HealthSystem, Evanston, Illinois, United States of America; University College London, United Kingdom

## Abstract

**Background:**

A multicenter study was conducted to evaluate the diagnostic accuracy (sensitivity and specificity) of the Verigene Gram-Positive Blood Culture Test (BC-GP) test to identify 12 Gram-positive bacterial gene targets and three genetic resistance determinants directly from positive blood culture broths containing Gram-positive bacteria.

**Methods and Findings:**

1,252 blood cultures containing Gram-positive bacteria were prospectively collected and tested at five clinical centers between April, 2011 and January, 2012. An additional 387 contrived blood cultures containing uncommon targets (e.g., *Listeria* spp., *S. lugdunensis*, *vanB*-positive Enterococci) were included to fully evaluate the performance of the BC-GP test. Sensitivity and specificity for the 12 specific genus or species targets identified by the BC-GP test ranged from 92.6%–100% and 95.4%–100%, respectively. Identification of the *mecA* gene in 599 cultures containing *S. aureus* or *S. epidermidis* was 98.6% sensitive and 94.3% specific compared to cefoxitin disk method. Identification of the *vanA* gene in 81 cultures containing *Enterococcus faecium* or *E. faecalis* was 100% sensitive and specific. Approximately 7.5% (87/1,157) of single-organism cultures contained Gram-positive bacteria not present on the BC-GP test panel. In 95 cultures containing multiple organisms the BC-GP test was in 71.6% (68/95) agreement with culture results. Retrospective analysis of 107 separate blood cultures demonstrated that identification of methicillin resistant *S. aureus* and vancomycin resistant *Enterococcus* spp. was completed an average of 41.8 to 42.4 h earlier using the BC-GP test compared to routine culture methods. The BC-GP test was unable to assign *mecA* to a specific organism in cultures containing more than one *Staphylococcus* isolate and does not identify common blood culture contaminants such as *Micrococcus*, *Corynebacterium*, and *Bacillus*.

**Conclusions:**

The BC-GP test is a multiplex test capable of detecting most leading causes of Gram-positive bacterial blood stream infections as well as genetic markers of methicillin and vancomycin resistance directly from positive blood cultures.

*Please see later in the article for the Editors' Summary*

## Abstract 2

Nathan Ledeboer and colleagues assess the performance of a diagnostic platform for detecting Gram-positive bacteria in blood cultures, an important step in the diagnosis and treatment of patients with sepsis.

*Please see later in the article for the Editors' Summary*

## Editors' Summary

**Background:**

Severe sepsis—a life-threatening condition that is usually is triggered by a bacterial infection of the bloodstream—is a major global cause of illness and death. In the US alone, sepsis causes up to 500,000 hospital admissions and more than 250,000 deaths a year. Normally, when microbes enter the human body, the immune system efficiently kills the invaders. In sepsis, the immune system goes into overdrive and the chemicals it releases into the blood to combat the infection trigger widespread inflammation, which leads to the formation of small blood clots and leaky blood vessels that impair the flow of blood to vital organs. In the most severe cases, multiple organs fail and the patient dies. Anyone can get sepsis but people with weakened immune systems, the elderly, and the very young are most vulnerable. Symptoms of sepsis include fever, chills, rapid breathing, and a fast heart rate. In its early stages, sepsis can be treated with antibiotics alone, but people with severe sepsis need to be admitted to an intensive care unit where their vital organs can be supported while the infection is treated.

**Why Was This Study Done?:**

The outcome of sepsis is affected by many factors, but fast, accurate identification of the bacterial infection and determination of its antibiotic susceptibility is essential to ensure that patients receive appropriate antibiotics. Laboratory diagnosis of bloodstream infections currently requires incubation of blood samples in a liquid medium (broth) followed by growth on solid media to identify the bacteria and to test for antibiotic sensitivity. It takes about 3 days after a positive broth culture is obtained to complete this process during which time patients are treated with broad spectrum antimicrobials, which may be ineffective. In this study, the researchers evaluate the sensitivity (a test's ability to identify patients who are positive for a specific condition) and specificity (a test's ability to identify patients who do not have a specific condition) of the Verigene BC-GP test. This multiplex, automated microarray-based nucleic acid test has been developed to directly and simultaneously identify 12 species of Gram-positive bacteria (the commonest cause of bacterial bloodstream infections) and three antibiotic resistance determinants in blood culture broths that contain Gram-positive bacteria.

**What Did the Researchers Do and Find?:**

The researchers used the Verigene BC-GP test and reference culture methods to identify the bacterial species and antibiotic resistance determinants present in 1,252 blood cultures containing Gram-positive bacteria collected at five US clinical centers and in 387 contrived blood cultures that contained bacterial species rarely found in the bloodstream. Compared to the reference culture method, the sensitivity of the Verigene BC-GP test for the bacterial species included in the test ranged from 92.6% to 100%; its specificity was 94.5%–100%. Identification of the *mecA* gene (a genetic marker for methicillin resistance) in cultures that contained *Staphylococcus aureus* or *S. epidermis* (Gram-positive bacteria that are often methicillin resistant) was 98.6% sensitive and 94.3% specific. Identification of the *vanA* gene (a genetic marker for vancomycin resistance) was 100% sensitive and specific in cultures containing *Enterococcus* species. Only 7.5% of single-organism cultures contained Gram-positive bacteria not present in the Verigene BC-GP test panel. Importantly, results obtained with the new test agreed those obtained with reference culture methods in three-quarters of cultures that contained more than one bacterial species. Finally, in a retrospective analysis of turn-around-time, identification of methicillin-resistant *S. aureus* and vancomycin-resistant *Enterococcus* species with the Verigene BC-GP test was about 42 hours faster than with reference methods.

**What Do These Findings Mean?:**

These findings show that the Verigene BC-GP test is capable of accurately identifying most leading causes of bloodstream infection with Gram-positive bacteria. Moreover, they show that the test can detect genetic markers of methicillin and vancomycin resistance directly from positive blood cultures, although they also reveal that the test cannot assign *mecA* positivity to a specific organism in a mixed culture, a finding that may mean that some patients are treated with unnecessary antibiotics. Overall, the researchers conclude that the Verigene BC-GP test has the potential to markedly reduce the turn-around-time for reporting bacterial identification from positive blood cultures. Its use should, therefore, improve the care of patients with sepsis by allowing physicians to prescribe appropriate antibiotics much earlier than is currently possible.

**Additional Information:**

Please access these Web sites via the online version of this summary at http://dx.doi.org/10.1371/journal.pmed.1001478.

- The US National Institute of General Medical Sciences has a fact sheet on sepsis
- The UK National Health Service Choices website has information about sepsis
- The Sepsis Alliance, a US not-for-profit organization, provides information about sepsis for patients and their families, including personal stories about sepsis
- The not-for profit UK Sepsis Trust is another useful source of information about sepsis that includes patient stories
- Wikipedia has a page on Gram-positive bacteria (note: Wikipedia is a free online encyclopedia that anyone can edit; available in several languages)
- Medlineplus provides links to additional resources about sepsis and about bacterial infections (in English and Spanish)

## Introduction

Sepsis resulting from bacterial bloodstream infection (BSI) is a serious condition that results in up to 500,000 hospitalizations per year and accounts for 11% of intensive care unit (ICU) admissions in the United States [1],[2]. Underscoring the significance of these infections is a mortality rate of 25% to 80% in critically ill patients [1],[3],[4]. The most prevalent causes of bacterial BSI are the Gram-positive bacteria, which account for 52% to 77% of bacterial sepsis [2],[5]. Among Gram-positive organisms, coagulase negative *Staphylococcus* spp. (CoNS) are most commonly isolated followed by *S. aureus* and *Enterococcus* spp. [5],[6]. *Streptococcus pneumoniae* are less frequently isolated but are of specific concern for patients with pneumococcal pneumonia [5]. BSIs carry a high monetary cost for the ill patient and also present a resource burden to the health care facility. In comparisons of patients admitted to hospital ICUs, those acquiring a BSI spent an additional 8 d in the ICU and 24 d in the hospital at an added cost of US$36,000 to US$40,000 per patient [4],[7].

The outcome of BSI can be affected by numerous factors including patient age, number, and type of co-morbidities, and time to effective antibiotic therapy [5],[8]. Indeed, effective antibiotic therapy has been independently correlated with positive outcome following culture confirmation of BSI [8],[9]. Kumar et al. report a 7.6% mean decrease in survival for every hour effective antibiotic therapy is delayed following the onset of sepsis-related hypotension [8]. Similarly, Bauer et al. demonstrated a reduction in length of ICU stay of 6.2 d and an overall savings of US$21,000 per septic episode by accurately identifying and differentiating *S. aureus*, methicillin-resistant *S. aureus* (MRSA), and coagulase negative staphylococci directly from positive blood cultures [10]. These savings were attributed, in part, to timely administration of appropriate antimicrobial therapy based on rapid laboratory results.

The current mainstay of laboratory diagnosis for BSI is broth-based culture of patient blood samples using a continuous monitoring blood culture system. Upon culture positivity, a primary Gram stain is performed and a portion of the broth culture is inoculated to solid media. Solid media subcultures require 18 to 48 h of incubation prior to biochemical testing of isolates to reach a definitive bacterial identification. Subsequent antibiotic susceptibility testing requires an additional 12–24 h for final result. This extended delay between confirmation of BSI, final identification, and susceptibility results leaves the clinician with little actionable information during a critical phase of infection. In response, patients are routinely treated with empiric broad spectrum antimicrobials that in some instances may be ineffective [11]. For this reason, a variety of technologies have been employed to shorten the window between blood culture positivity and the availability of results useful in guiding therapy.

Molecular methods, fluorescent *in situ* hybridization (FISH), and more recently matrix assisted laser desorption ionization time-of-flight mass spectrometry (MALDI-ToF MS) have all been used to rapidly identify a variety of organisms directly from positive blood cultures [12]–[19]. These methods all significantly reduce turn-around-time (TAT) compared to routine culture, delivering results in as little as 30–60 min following blood culture positivity. Sensitivity and specificity of these methods are generally high, reaching >90% for each method. Unfortunately, current molecular methods and FISH are limited to the detection of one or few specific targets. MALDI-ToF MS has the potential to identify nearly any bacterial species present in a blood culture; however, acceptable confidence score results were obtained for only 67% to 80% of cultures containing Gram-positive bacteria when using standard protocols [12]–[14]. Further, differentiation between *S. pneumoniae* and *S. mitis* using MALDI-ToF MS remains an active area of investigation [20]–[22]. These shortcomings have been addressed primarily through optimization of spectral analysis software and modification of scoring thresholds [12],[20]–[22].

In this study we evaluate the microarray based Verigene Gram-Positive Blood Culture test (BC-GP) (Nanosphere) for the identification of 12 Gram-positive bacterial targets and three genetic markers of antibiotic resistance directly from positive blood culture broths. This test was intended for analysis of positive blood cultures confirmed to contain Gram-positive organisms upon primary Gram stain of the broth. The specific targets identified by the BC-GP test include *Staphylococcus spp*., *S. aureus*, *S. epidermidis*, *S. lugdudensis*, *Streptococcus spp*., *S. pyogenes*, *S. agalactiae*, *S. anginosus* group, *S. pneumoniae*, *E. faecalis*, *E. faecium*, and *Listeria spp*. as well as the *mecA*, *vanA*, and *vanB* genes. We report sensitivity and specificity of the BC-GP test compared to routine culture methods for 1,252 prospectively collected blood cultures including 95 polymicrobial cultures obtained at five medical centers using BACTEC (BD) and BacT/ALERT (bioMérieux) blood culture systems.

## Materials and Methods

### Collection of Blood Culture Broths

A total of 1,252 positive blood culture broths were prospectively and consecutively collected at five clinical centers located in Wisconsin, New York, Illinois, Texas, and Ohio in accordance with site-specific institutional review board (IRB)-approved study protocols. Specimens were enrolled in this study from April, 2011 to January, 2012. Compliance criteria for this study included blood cultures using BACTEC Plus Aerobic/F (BD) and BacT/ALERT FA FAN (bioMérieux) aerobic blood culture medium. Blood culture broths were eligible for study enrolment if they contained Gram-positive cocci or bacilli on primary Gram stain and could be tested on the BC-GP within 12 h of broth positivity (held at room temperature). Only one positive blood culture per patient was allowed to avoid redundancy of enrolled samples (Text S1).

### Verigene Gram-Positive Blood Culture Test

The BC-GP test is a sample to result system consisting of a sample processor (SP) and microarray reader. This test was run by laboratory technologists who were trained by the manufacturer on how to perform the test. A single use extraction tray was inserted into the SP and a 350 µl aliquot of positive blood culture broth containing Gram-positive organisms was transferred to the sample well within the extraction tray. Nucleic acid was extracted from blood culture samples using magnetic bead-based extraction and reagents contained in the extraction tray. No amplification of nucleic acid is performed. Purified nucleic acid was automatically hybridized to complementary nucleic acid capture probes immobilized on a glass microarray slide within the SP. Capture probes for each BC-GP test target are present in triplicate on the array. Detection of target sequence relies on hybridization of a second, nanoparticle-conjugated, detection probe. This method allows up to 1,000-fold greater sensitivity than fluorescent probes and requires comparatively simple excitation and detection optics [23],[24]. Automated sample processing (nucleic acid extraction and array hybridization) in the SP takes 2.5 h. Reading of the array is conducted in the Verigene Reader following processing and takes 30 to 60 sec. Tests generating indeterminate results (e.g., internal control failure, extraction control failure, variation in target signal, high background signal) were repeated a single time. A total of 64 cultures generated an indeterminate result upon initial analysis resulting in an initial call rate of 94.9% (1,188/1,252). Of these, 82.8% (53/64) were resolved following a single retest for a final call rate of 99.1%.

### Reference Culture Method

Reference method testing was conducted by trained laboratory technologists (Figure S1; Text S1). To avoid bias, technologists conducting reference method testing were blinded to results obtained using the BC-GP test. Broth from blood cultures that were analyzed using the BC-GP test was inoculated to Trypticase Soy Agar with 5% Sheep Blood (TSA, BBL 221261 or equivalent, BD) and incubated at 35°C for 48 h. Following 24 and 48 h incubation, two glycerol stocks (Trypticase Soy Broth with 20% glycerol, BD) were made from each unique colony type present on the plate. One stock was retained by the test lab and the other was shipped on dry ice to a third party reference lab (Dynacare Laboratories). Glycerol stocks received by the reference laboratory were thawed, inoculated to TSA, and incubated 24 h at 35°C in 5% CO_2_. Following incubation, an isolated colony(s) representative of each colony type present on the plate were passed a second time onto TSA and incubated 24–48 h at 35°C. Glycerol stocks were made from resulting bacterial growth and each bacterial isolate was identified according to a reference culture flow chart using biochemical tests (Figure S1; Text S1). Briefly, *S. aureus* was identified on the basis of a positive catalase reaction, observation of beta-hemolysis on TSA, and a positive latex agglutination test. CoNS were identified similarly; however CoNS could demonstrate either beta- or gamma -hemolysis on TSA coupled with a negative latex agglutination test. *S. lugdunensis* were identified with the addition of a positive test for ornithine decarboxylase and L-pyrrolidonyl arylamidase (PYR) activity. All CoNS were identified to species using Vitek 2 Gram-positive identification card (GPID). All isolates identified to species level as *S. aureus* or *S. epidermidis* were tested for resistance to methicillin using a cefoxitin disk diffusion assay according to CLSI guidelines [25]. *Micrococcus* spp. were identified based upon a positive catalase test, yellow colored colony, and a positive microdase disk test. *Listeria* spp. were identified using Gram stain (Gram-positive bacilli), the presence of “soft” beta-hemolysis on TSA, a positive catalase reaction, and Vitek GPID. *Enterococcus* spp. were identified by a negative catalase test and positive reactions for PYR and lucine aminipepdidase (LAP) along with the ability to grow in the presence of 6.5% NaCl in TSB broth and form black precipitate when cultured on bile esculin agar. *E. faecalis* and *E. faecium* were differentiated by the ability to utilize arabinose [*E. faecium* (+), *E. faecalis* (−)]. All isolates of *E. faecium* or *E. faecalis* were tested for resistance to vancomycin using Etest vancomycin (bioMérieux). Other species of *Entercoccus* were identified using Vitek 2 GPID. *Streptococcus* spp. were identified based upon negative catalase and PYR reactions combined with a positive LAP result. Colonies demonstrating beta hemolysis were typed using latex agglutination tests for Lancefield antigen types A (*S. pyogenes*) and B (*S. agalactiae*). If negative for type A and B antigens, *S. anginosus* group identification was made on the basis of positive Voges-Proskauer and arginine fermentation tests. *S. pneumonia*e was differentiated from other Streptococci on the basis of susceptibility to optochin (disk diffusion test) and bile solubility. Additionally, all *Streptococcus* spp. were identified using Vitek 2 GPID (bioMérieux).

### Contrived Cultures

A challenge set of 387 contrived (n = 213) or previously characterized (n = 174) blood cultures was constructed to test BC-GP targets that are rarely encountered in clinical laboratories (e.g., *Listeria* spp., *vanB*, etc.). For contrived cultures, characterized clinical isolates were seeded into BACTEC Plus Aerobic/F blood broths supplemented with 10 ml fresh whole blood. Broths were incubated in a BACTEC continuous monitoring blood culture instrument until positivity. A 1 ml aliquot of the positive broth culture was removed, frozen, blinded, and shipped to one of the five clinical test sites for analysis using the BC-GP. Reference culture identification was conducted as described above. Since the BC-GP *Listeria* target is a genus only target various species were used in construction of the challenge set including *L. monocytogenes* (*n = *21), *L. innocua* (*n = *7), *L. ivanovii* (*n = *2), *L. seeligeri*, and *L. welshimeri*.

### Nucleic Acid Sequencing and Discrepant Analysis

All *E. faecalis* and *E. faecium* isolates testing positive for *vanA* or *vanB* by BC-GP and/or demonstrating phenotypic resistance to vancomycin were sequenced by a third-party service laboratory (ACGT, Inc.) to confirm the presence of *vanA/vanB*. Extracted DNA was amplified with either *vanA-* or *vanB*-specific PCR primers and run on an agarose gel to observe the presence of a specific amplification product. Specific PCR-amplified products were then sequenced with the appropriate specific primers and the raw sequence data was subjected to PHRED score analysis. Only PHRED 30 quality sequences were collected and aligned to determine the specific *van* allele variant.

Procedures used for the in-house PCR assay to detect *mecA* were those described by Paule et al., including DNA extraction, primers, real-time PCR conditions, and melt analysis to confirm amplicon identity [26]. Ribosomal 16S gene sequencing method and primer design was performed as described by Clarridge et al. and as found in CLSI MM18-A [27]. PCR and sequencing methods followed protocols for *rpoB* gene amplification and sequencing described by Khamis et al. [28]. 16S rRNA nucleotide sequences were analyzed and interpreted using the MEGA software [29] and BLAST algorithm (http://blast.ncbi.nlm.nih.gov/Blast.cgi).

### Assessment of Time to Identification Using BC-GP and Routine Culture Methods

A retrospective comparison of time to final identification and susceptibility result was conducted using a collection of 107 consecutive blood culture broths with a positive Gram stain for Gram-positive organisms. This additional study was conducted following laboratory validation and implementation of the BC-GP test for clinical use at a single study site (Medical College of Wisconsin, Milwaukee) in accordance with a separately IRB approved protocol allowing for collection of laboratory values including time of culture positivity, standard of care identification and susceptibility results, and time of final culture report. These cultures were not part of the 1,252 cultures prospectively collected for evaluation of the performance of the BC-GP test. Cultures were selected on the basis of a positive Gram-stain (Gram-positive bacteria present). Only the initial positive culture from a patient was analyzed using the BC-GP test. Time zero was defined as the time at which the primary culture Gram stain result was reported to appropriate medical staff responsible for care of the patient. Upon completion of the BC-GP test, the medical technologist directly notified the physician responsible for patient care by telephone with a “preliminary identification.” The time to result for the BC-GP test was defined as the time that the result was reported to the physician. For culture based identification and susceptibility, the time at which final results were entered into the laboratory information management system (available to medical staff) was utilized to determine total time to identification/susceptibility. The BC-GP test was set up following primary Gram-stain confirmation of Gram-positive bacteria in broth culture. BC-GP test results were available within in 2 h of test initiation. Standard culture method involved subculture of the positive broth to TSA and chocolate agar (Remel) and incubation at 35°C for 18–24 h. Resulting colonies that tested positive for catalase activity were subjected to a *Staphylococcus* latex agglutination test for preliminary differentiation of *S. aureus* from other *Staphylococcus* species. Full identification and susceptibility testing of isolates was conducted using the BD Phoenix system (BD). Colonies preliminarily identified as coagulase negative *Staphylococcus* species were only subjected to full identification and susceptibility if they were present in multiple culture from the same patient.

### Statistical Analysis

Sensitivity and specificity were calculated using standard methods. Ninety-five percent confidence intervals were calculated using binomial expansion. *p*-Values were calculated using matched two-tailed t-test.

## Results

### Prospectively Collected Monomicrobial Cultures

A total of 1,157 prospectively collected blood cultures contained a single organism as determined by reference culture method. The majority of these contained *Staphylococcus* spp., which accounted for 73.0% (845/1,157) of monomicrobial cultures, followed by *Streptococcus* spp., 11.7% (135/1,157), *E. faecalis* or *faecium*, 7.4% (86/1,157), and *Listeria* spp., 0.3% (3/1,154). Only 7.5% (87/1,157) of cultures contained organisms not included on the BC-GP panel. The majority of these cultures were positive for *Micrococcus* spp., *Corynebaterium* spp., *Kocuria* spp., and *Bacillus* spp.

Sensitivity of the *Staphylococcus* genus target was a combined 99.4% (840/845) (Table 1). Five cultures categorized as false negative were resulted as “not detected” by the BC-GP test. These cultures all contained CoNS including one methicillin susceptible *S. epidermidis* (MSSE), one methicillin resistant *S. epidermidis* (MRSE), two *S. hominis*, and one *S. haemolyticus*. Specificity of the *Staphylococcus* genus target was 99.7% (310/311). The false positive result was reported as “*Staphylococcus* spp.” by the BC-GP test but the reference method culture identified the isolate as *Aerococcus viridans*. The *S. aureus* species target was 99.7% (317/318) sensitive and 100% specific. The single false negative was identified as methicillin susceptible *S. aureus* (MSSA) by culture method but “*Staphylococcus* spp.” only by BC-GP. *S. epidermidis* was identified with a sensitivity of 96.5% (272/282) and specificity of 98.9% (864/874). Of ten false negative *S. epidermidis* results, seven were reported as “*Staphylococcus* spp.” only and three were reported as “not detected” by the BC-GP test. Nucleic acid sequencing used to resolve discrepant results confirmed the identification of *S. epidermidis* in six of seven cultures reported as “*Staphylococcus* spp.” (i.e., false negative). The ten false positive cultures with a BC-GP test result of “*S. epidermidis*” contained one *S. intermedius*, two each *S. capitis*, *S. hominis*, and “CoNS” and three *S. haemolyticus*. Nucleic acid sequencing supported the BC-GP test result (*S. epidermidis*) in five of ten discrepant cultures, raising the final specificity to 99.4% (864/869). Identification of *S. lugdunensis* was 100% sensitive and specific, however only eight prospectively collected cultures contained this organism.

**Table 1:** Detection of *Staphylococcus* spp. in prospectively collected monomicrobial blood cultures by Verigene BC-GP (*n = *1,157).

Target	Site	TP	FP	TN	FN	Total^a^	Sensitivity (CI)^b^	Specificity (CI)
***Staphylococcus*** ** spp.**	A	97	0	36	0	133	100% (96–100)	100% (96–100)
	B	196	0	58	0	254	100% (98–100)	100% (93–100)
	C	83	0	43	0	126	100% (95–100)	100% (91–100)
	D	243	1	104	5	353	98.0% (95–99)	99.1% (94–99)
	E	221	0	70	0	291	100% (98–100)	100% (94–100)
	**Total**	**840**	**1**	**310**	**5**	**1,157**	**99.4% (98**–**100)**	**99.7% (98**–**100)**
***S. aureus***	A	41	0	92	0	133	100% (91–100)	100% (96–100)
	B	80	0	173	1	254	98.8% (93–99)	100% (97–99)
	C	31	0	95	0	126	100% (88–100)	100% (96–100)
	D	102	0	251	0	353	100% (96–100)	100% (98–100)
	E	63	0	228	0	291	100% (98–100)	100% (94–100)
	**Total**	**317**	**0**	**838**	**1**	**1,157**	**99.7% (98**–**100)**	**100% (99**–**100)**
***S. epidermidis***	A	37	0	94	2	133	94.9% (90–100)	100% (96–100)
	B	62	1	190	1	254	98.4% (91–99)	99.5% (97–99)
	C	29	2	94	1	126	96.7% (82–99)	97.9% (92–99)
	D	60	2	285	6	353	90.9% (81–96)	99.3% (97–99)
	E	84	5	202	0	291	100% (95–100)	97.6% (94–99)
	**Total**	**272**	**10** c	**864**	**10** d	**1,157**	**96.5% (93**–**98)**	**98.9% (97**–**99)**
***S. lugdunensis***	A	1	0	132	0	133	100% (2–100)	100% (97–100)
	B	1	0	253	0	254	100% (2–100)	100% (98–100)
	C	0	0	126	0	125	NA	100% (97–100)
	D	2	0	351	0	353	100% (15–100)	100% (98–100)
	E	4	0	287	0	291	100% (39–100)	100% (98–100)
	**Total**	**8**	**0**	**1,148**	**0**	**1,157**	**100% (63**–**100)**	**100% (99**–**100)**

a Total number of cultures with reported result.

b 95% confidence interval.

c Nucleic acid sequencing confirmed the identification of *S. epidermidis* in five of ten cultures.

d Nucleic acid sequencing confirmed the identification of *S. epidermidis* in six of ten cultures.

FN, false negative; FP, false positive; TN, true negative; TP, true positive.

*Streptococcus* spp. were identified with 94.8% (128/135) sensitivity and 99.6% (1,018/1,022) specificity in prospectively collected cultures (Table 2). The seven false negative results were all reported as “not detected” by the BC-GP test and included six cultures containing alpha hemolytic streptococci (two *S. mitis*, *S. mutans*, *S. sanguinis*, *S. salivarius*, and “viridans group streptococcus”). The remaining false negative “*Streptococcus* spp.” result was a culture containing *S. agalactiae*. Four cultures incorrectly reported as positive for “*Streptococcus* spp.” contained *Lactococcus garvieae* (two), *Granulicatella adjacens*, and *Lactococcus* spp. Sensitivity for identification of *S. pyogenes* (9/9), *S. pneumoniae* (23/23), and *S. anginosus* group (8/8) species targets were all 100%. Five cultures incorrectly reported as “*S. pneumoniae*” by the BC-GP test were found to contain viridans group streptococci, including four of five that contained *S. mitis* as determined by the reference method.

**Table 2:** Detection of *Streptococcus* spp. in prospectively collected monomicrobial blood cultures by Verigene BC-GP (*n = *1,157).

Target	Site	TP	FP	TN	FN	Total^a^	Sensitivity (CI)^b^	Specificity (CI)
***Streptococcus*** ** spp.**	A	11	0	122	0	133	100% (71–100)	100% (97–100)
	B	20	0	234	0	254	100% (83–100)	100% (98–100)
	C	24	0	99	3	126	88.9% (70–97)	100% (96–100)
	D	43	2	307	1	353	97.7% (87–99)	99.4% (97–99)
	E	30	2	256	3	291	90.9% (75–98)	99.2% (97–99)
	**Total**	**128**	**4**	**1,018**	**7**	**1,157**	**94.8% (89**–**97)**	**99.6% (99**–**100)**
***S. pyogenes***	A	1	0	132	0	133	100% (2–100)	100% (97–100)
	B	0	0	254	0	254	NA	100% (98–100)
	C	2	0	124	0	126	100% (15–100)	100% (97–100)
	D	3	0	350	0	353	100% (29–100)	100% (98–100)
	E	3	0	288	0	291	100% (29–100)	100% (98–100)
	**Total**	**9**	**0**	**1,148**	**0**	**1,157**	**100% (66**–**100)**	**100% (99**–**100)**
***S. agalactiae***	A	5	0	128	0	133	100% (47–100)	100% (97–100)
	B	9	0	245	0	254	100% (98–100)	100% (98–100)
	C	8	0	118	0	126	100% (63–100)	100% (96–100)
	D	9	0	344	0	353	100% (66–100)	100% (98–100)
	E	5	0	285	1	291	83.3% (35–99)	100% (98–100)
	**Total**	**36**	**0**	**1,120**	**1**	**1,157**	**97.3% (85**–**99)**	**100% (99**–**100)**
***S. anginosus*** ** gr.**	A	0	0	133	0	133	NA	100% (97–100)
	B	0	0	254	0	254	NA	100% (98–100)
	C	1	1	124	0	126	100% (2–100)	99.2% (95–100)
	D	2	2	349	0	353	100% (6–93)	99.4% (97–100)
	E	5	0	286	0	291	100% (47–100)	100% (98–100)
	**Total**	**8**	**3**	**1,146**	**0**	**1,157**	**100% (63–100)**	**99.7% (99–100)**
***S. pneumoniae***	A	0	1	132	0	133	NA	99.2% (95–100)
	B	2	0	252	0	254	100% (15–100)	100% (98–100)
	C	5	0	121	0	126	100% (47–100)	100% (97–100)
	D	9	4	340	0	340	100% (66–100)	98.8% (97–99)
	E	7	0	284	0	291	100% (59–100)	100% (98–100)
	**Total**	**23**	**5**	**1,129**	**0**	**1,157**	**100% (85–100)**	**99.6% (98–100)**

a Total number of cultures with reported result.

b 95% confidence interval.

FN, false negative; FP, false positive; TN, true negative; TP, true positive.

The BC-GP test does not contain a general genus target for *Enterococcus* spp., rather, it specifically identifies only *E. faecalis* and *E. faecium* species. Sensitivity for identification of these species was 94.8% (55/58) and 92.6% (26/28), respectively (Table 3). All five cultures categorized as false negative were reported as “not detected” by BC-GP but were positive for *E. faecalis* or *E. faecium* by the reference method. The single false positive result came from a culture reported as positive for both *E. faecalis* and *S. pyogenes* by BC-GP, however only *S. pyogenes* was recovered upon subculture and reference testing. Only three prospectively collected blood cultures contained *Listeria* spp., including two with *L. monocytogenes* and one with *L. innocua*. The BC-GP was 100% sensitive and specific for detection of these organisms.

**Table 3:** Detection of *E. faecalis*, *E. faecium*, and *Listeria* in prospectively collected monomicrobial blood cultures by Verigene BC-GP (*n = *1,157).

Target	Site	TP	FP	TN	FN	Total^a^	Sensitivity (CI)^b^	Specificity (CI)
***E. faecalis***	A	10	0	123	0	133	100% (69–100)	100% (97–100)
	B	11	0	242	1	254	91.7% (61–99)	100% (98–100)
	C	6	0	119	1	126	85.7% (42–99)	100% (96–100)
	D	16	1	335	1	353	94.1% (71–99)	99.7% (98–99)
	E	12	0	279	0	291	100% (73–100)	100% (98–100)
	**Total**	**55**	**1**	**1,098**	**3**	**1,157**	**94.8% (85–98)**	**99.9% (99–100)**
***E. faecium***	A	5	0	127	1	133	83.3% (35–99)	100% (97–100)
	B	9	0	245	0	254	100% (66–100)	100% (98–100)
	C	0	0	126	0	126	NA	100% (97–100)
	D	9	0	343	1	353	90.0% (55–99)	100% (98–100)
	E	3	0	288	0	291	100% (29–100)	100% (98–100)
	**Total**	**26**	**0**	**1,129**	**2**	**1,157**	**92.6% (76–99)**	**100% (99–100)**
***Listeria***	A	0	0	133	0	133	NA	100% (97–100)
	B	0	0	254	0	254	NA	100% (98–100)
	C	0	0	126	0	126	NA	100% (97–100)
	D	2	0	351	0	353	100% (15–100)	100% (98–100)
	E	1	0	290	0	291	100% (2–100)	100% (98–100)
	**Total**	**3**	**0**	**1,154**	**0**	**1,157**	**100% (29–100)**	**100% (99–100)**

a Total number of cultures with reported result.

b 95% confidence interval.

FN, false negative; FP, false positive; TN, true negative; TP, true positive.

### Identification of mecA, vanA, and vanB as Markers of Antibiotic Resistance

The BC-GP test identifies the *mecA* gene, which serves as an indicator of resistance to methicillin in *Staphylococcus* spp., and the *vanA* and *vanB* genes, which serve as markers of resistance to vancomycin in *E. faecalis* and *E. faecium*. Although *mecA* can be found in most species of *Staphylococcus*, the BC-GP only reports *mecA* positivity in *S. aureus* and *S. epidermidis*. Among the five clinical test sites, methicillin resistance in *S. aureus* and *S. epidermidis* ranged from 46.8% to 64.5% as determined by cefoxitin disk diffusion test. The BC-GP test was 98.6% (348/353) sensitive and 94.3% (232/246) specific for prediction of methicillin resistance based on *mecA* positivity when using cefoxitin disk test results as gold standard comparator (Table 4). The five false negative methicillin resistance results were noted for both *S. epidermidis* (3/5) and *S. aureus* (2/5). Four of these isolates were available for discrepant analysis using an alternative nucleic acid amplification test (NAAT) specific for *mecA*. All four isolates tested negative using the alternative NAAT, raising the post discrepant resolution sensitivity to 99.7% (348/349). False positive results were more common among *S. epidermidis* (13/14) compared to *S. aureus* (1/14). Discrepant analysis using an alternative NAAT was conducted and confirmed the presence of *mecA* in 8/14 of these isolates, raising the final post discrepant resolution specificity to 97.5% (232/238).

**Table 4:** Detection of resistance determinants *mecA, vanA, and vanB* in prospectively collected monomicrobial blood cultures by Verigene BC-GP (*n = *599 *S. aureus/S. epidermidis*, *n = *81 *E. faecalis/E. faecium*).

Target	Site	TP	FP	TN	FN	Total^a^	Sensitivity (CI)^b^	Specificity (CI)
***mecA*** c	A	41	1	36	0	78	100% (91–100)	97.3% (58–99)
	B	92	2	49	0	143	100% (96–100)	96.1% (86–99)
	C	29	3	30	0	62	100% (88–100)	90.9% (75–98)
	D	89	2	69	4	164	95.7% (89–98)	97.2% (90–99)
	E	97	6	48	1	152	99.0% (94–100)	88.9% (77–95)
	**Total**	**348**	**14** d	**232**	**5** e	**599**	**98.6% (96–99)**	**94.3% (90–96)**
***vanA*** f	A	4	0	11	0	15	100% (39–100)	100% (79–100)
	B	9	0	11	0	20	100% (66–100)	100% (71–100)
	C	0	0	6	0	6	NA	100% (54–100)
	D	10	0	15	0	25	100% (69–100)	100% (78–100)
	E	3	0	12	0	15	100% (29–100)	100% (73–100)
	**Total**	**26**	**0**	**55**	**0**	**81**	**100% (86–100)**	**100% (93–100)**
***vanB*** f, g	**Total**	**0**	**0**	**0**	**0**	**0**	**NA**	**NA**

a Total number of cultures with reported result.

b 95% confidence interval.

c *mecA* is reported only for cultures with positive *S. aureus* or *S. epidermidis* species targets. Inferred based upon resistance to cefoxitin using disk diffusion method.

d *mecA* specific PCR was positive for eight of 14 isolates. Final specificity of 97.5%.

e *mecA* specific PCR was negative for four of five isolates. Final sensitivity of 99.7%.

f vanA and vanB are reported only for cultures with positive *E. feacium* or *E. faecalis* targets.

g *vanB* was not detected in any prospectively collected cultures.

FN, false negative; FP, false positive; TN, true negative; TP, true positive.

Vancomycin resistance was observed in 55 of 81 *E. faecalis* or *E. faecium* isolates recovered from positive blood culture broths as determined by Etest vancomycin reference method. The BC-GP was 100% sensitive and specific, reporting a *vanA* positive result for all vancomycin resistant isolates. Phenotypic resistance and genotype (*vanA* or *vanB*) was confirmed by nucleic acid sequencing, which confirmed the presence of *vanA* sequence variant-2 in these isolates (Table S2).

### Polymicrobial Blood Cultures

Prospectively collected blood cultures included 95/1,252 (7.6%) that were found to contain more than one organism, i.e., polymicrobial, when tested by reference culture method. The majority of these (88/95) contained two different organisms while seven cultures contained three organisms (Table 5). Of the 95 polymicrobial cultures, 53 (55.8%) were composed of CoNS in addition to a second or third organism. The BC-GP was in complete agreement with the reference culture method for 68/95 (71.6%) cultures. False negative BC-GP results were far more common (*n = *26) than false positive results (*n = *3) among discordant cultures. The most commonly missed target was CoNS (*n = *19) including 12 *S. epidermidis*, one *S. lugdunensis*, and six other cultures containing various CoNS species in addition to a second or third organism. *S. aureus* was missed in two polymicrobial cultures, both of which also contained *Enterococcus* which was correctly identified by BC-GP. The remaining five false negative results were *Streptococcus* spp. not detected by BC-GP that were isolated from cultures also containing CoNS or organisms not on the BC-GP panel (e.g., *Rothia*, *Leuconostoc*).

**Table 5:** Detection of Gram-positive identification and resistance determinants from polymicrobial blood cultures by Verigene BC-GP (*n = *95).

Culture Results	Total	Verigene BC-GP Results		
		Match Culture	Target Missed (False Negative)	Additional Target (False Positive)
**Two organisms**				
One of two on BC-GP panel	20	16/20	3 *Streptococcus* spp.	1 *Staphylococcus* spp.
Neither on BC-GP panel	4	4/4		
CoNS + CoNS^a^	11	11/11		
*S. epidermidis* + CoNS	24	19/24	5 *S. epidermidis*	
*S. aureus* + CoNS	3	3/3		
*S. lugdunensis* + CoNS	1	0/1	1 *S. lugdunensis*	1 *S. epidermidis*
*S. lugdunensis* + *Listeria* b	1	1/1		
*S. epidermidis* + *S. pyogenes*	1	0/1	1 *S. epidermidis*	
*S. epidermidis* + *S. agalactiae*	1	0/1	1 *S. epidermidis*	
*S. aureus* + *S. agalactiae*	1	1/1		
*Enterococcus* c + CoNS	9	5/9	4 CoNS	
*Enterococcus* + *S. aureus*	2	0/2	2 *S. aureus*	
*Enterococcus* + *S. epidermidis*	5	1/5	4 *S. epidermidis*	
*Enterococcus* + *S. agalactiae*	1	1/1		
*Streptococcus.* spp. + CoNS	3	1/3	2 *Streptococcus spp.*	
*Streptococcus* spp. (×2)	1	1/1		
**Three organisms**				
*E. faecalis* + *E. faecium* + *GNR* d	1	1/1		
*E. faecalis* + *Bacillus* e + *GNR*	1	0/1		1 *Streptococcus* spp.
*Streptococcus spp. (×2)* + *GNR*	2	2/2		
*E. faecalis* + *CoNS* + *Yeast*	1	0/1	1 CoNS	
*S. aureus* + *S. epidermidis* + *Dermacoccus*	1	1/1		
*S. epidermidis* + *CoNS* + *CoNS*	1	0/1	1 *S. epidermidis*	
**Total**	95	68/95		

a Coagulase negative *Staphylococcus* species, not including *S. epidermidis* and *S. lugdunensis*.

b Contrived culture.

c *E. faecalis* or *E. faecium*.

d *Gram-negative rod*.

e *Bacillus* spp. not *anthracis*.

### Contrived Blood Cultures

Only 4/387 contrived cultures yielded a false negative identification result by BC-GP (Table 6). Two cultures contained *S. pyogenes* by reference culture method, one of which was reported as “not detected” and the other which was reported as “*Streptococcus* spp.” by BC-GP. This translates to a sensitivity of 99.3% (154/155) for *Streptococcus* spp. and 97.1% (66/68) for *S. pyogenes* among contrived cultures. The remaining false negative result was a culture containing *E. faecalis* that was misidentified as *E. faecium* by BC-GP. All other bacterial targets demonstrated 100% sensitivity. The specificity of the identification for all targets was also high in the contrived culture study. BC-GP reported positive results that were not confirmed by reference culture method in only 5/387 contrived cultures. These included a culture containing *S. xylosus* that was incorrectly identified as *S. lugdunensis* by BC-GP, an *E. faecium* that was incorrectly identified as *E. faecalis*, a culture containing only *L. monocytogenes* that was reported as positive for *Listeria* and *S. anginosus* (false positive for “*Staphylococcus* spp.” and *S. anginosus* group” targets), and a culture containing only *E. faecalis* that was reported as positive for *E. faecalis* and “*Staphylococcus* spp.”.

**Table 6:** Detection of Gram positive identification and resistance determinants from contrived blood cultures by Verigene BC-GP (*n = *387).

Target	TP	FP	TN	FN	Total^a^	Sensitivity (CI)^b^	Specificity (CI)
**Identification**							
*Staphylococcus* spp.	54	1	332	0	387	100% (93–100)	99.7% (98–100)
*S. aureus*	10	0	377	0	387	100% (69–100)	100% (99–100)
*S. epidermidis*	4	0	383	0	387	100% (39–100)	100% (99–100)
*S. lugdunensis*	31	1	355	0	387	100% (88–100)	99.7% (98–100)
*Streptococcus* spp.	154	1	231	1	387	99.3% (69–100)	99.6% (97–100)
*S. pyogenes*	66	0	319	2	387	97.1% (97–99)	100% (98–100)
*S. agalactiae*	36	0	351	0	387	100% (90–100)	100% (98–100)
*S. anginosus* gr.	26	1	360	0	387	100% (86–100)	99.7% (98–100)
*S. pneumoniae*	21	0	366	0	387	100% (83–100)	100% (99–100)
*E. faecalis*	32	0	354	1	387	97.0% (84–99)	100% (98–100)
*E. faecium*	77	1	309	0	387	100% (95–100)	99.7% (98–100)
*Listeria*	33	0	354	0	387	100% (89–100)	100% (98–100)
**Resistance**							
*mecA* c	9	0	5	0	14	100% (66–100)	100% (47–100)
*vanA* d	45	3	62	0	110	100% (92–100)	95.4% (87–99)
*vanB* d	39	0	70	1	110	97.5% (89–99)	100% (94–100)

a Total number of cultures with reported result.

b 95% confidence interval.

c *mecA* is reported only for cultures with positive *S. aureus* or *S. epidermidis* species targets.

d *vanA* and *vanB* are reported only for cultures with positive *E. feacium* or *E. faecalis* targets.

FN, false negative; FP, false positive; TN, true negative; TP, true positive.

Identification of genetic resistance determinants *mecA*, *vanA*, and *vanB* was also assessed using contrived cultures. Eighty-five cultures contained strains of *E. faecalis* or *E. faecium* that demonstrated resistance to vancomycin by Etest. The BC-GP demonstrated an overall sensitivity of 98.8% (84/85) for the identification of *vanA* (100%, 45/45) or *vanB* (97.5%, 39/40) in these isolates. All *vanA* genes were confirmed as sequence variant-2 using nucleic acid sequencing. Strains that were positive for *vanB* were more diverse in sequence including 25 variant-6, 11 variant-14, three variant-8, and one variant-10 alleles. The single strain false negative for *vanB* contained a sequence variant-6 allele.

### Time to Identification

The difference in time between reporting of results using the BC-GP test and final identification and antimicrobial susceptibility results was examined for 107 positive blood culture broths containing Gram-positive bacteria. Of these, 76 (71.0%) were reported as Gram-positive cocci in clusters (*Staphylococcus* spp., 68; *Micrococcus* spp., seven; *Rothia* spp., one). Results for cultures containing *S. aureus* (MRSA or MSSA) were available an average of 42.2 h (range 31 to 68 h) before final identification and susceptibility results were reported using routine culture and susceptibility testing methods (Table 7). Only eight of 43 cultures containing CoNS met criteria for full identification and susceptibility testing (>one set of blood cultures containing CoNS with different morphology), however preliminary identification of CoNS using culture methods was reported an average of 27.5 h (range 14 h to 48 h) after availability of the BC-GP test result. Among 22 positive cultures with a Gram stain indicating the presence of Gram-positive cocci in chains (*Streptococcus* spp., 12; *Enterococcus* spp., ten), BC-GP results were available an average of 53.4 h (range 31 to 127 h) before routine culture results. Importantly, this includes the identification of vancomycin-resistant *Enterococcus* (VRE), which was available an average of 44.4 h (range 33 to 55 h) before final results using routine culture and susceptibility testing methods.

**Table 7:** Difference in time to final identification and antimicrobial susceptibility report (*n = *107).

Culture Result	*n*	Verigene Agreement (%)	ΔTime to Result^a^		
			Average (h)	Range (h)	*p*-Value
***Staphylococcus***					
MRSA	9	9/9 (100)	41.8	32–68	*p*<0.001
MSSA	16	16/16 (100)	42.4	31–35	*p*<0.001
CoNS^b^	43^b^	40/43 (93.0)^c^	46.9^d^	34–67^d^	*p*<0.001
***Streptococcus***					
*S. pneumoniae*	3	3/3 (100)	43.7	41–46	*P* = 0.001
Viridians group	9	8/9 (88.9)^e^	74.8^f^	41–127^f^	*p* = 0.042
***Enterococcus***					
VRE	5	5/5 (100)	44.4	35–55	*p*<0.001
VSE	4	4/4 (100)	42.2	31–71	*p* = 0.024
**Polymicrobial**					
*S. lugdensis, S. epidermidis*	1	0/1 (0)^g^	60	n/a	n/a
*S. aureus, S. epidermidis*	1	1/1 (100)	43	n/a	n/a
**Not on BC-GP panel**					
*Micrococcus spp.*	7	n/a	n/a	n/a	n/a
*Corynebacterium* spp.	3	n/a	n/a	n/a	n/a
*Bacillus spp.*	2	n/a	n/a	n/a	n/a
Other	4	n/a	n/a	n/a	n/a

a Difference in time between BC-GP result and final culture-based identification and susceptibility results.

b Includes 26 *S. epidermidis*.

c Three cultures reported as “Not Detected” by BC-GP.

d Based on eight cultures that met criteria for full identification and susceptibility testing. Average time to “CoNS” identification only was 27.5 h (range 14 h to 48 h) earlier using BC-GP, *n = *35.

e One culture reported as *S. pneumoniae* by BC-GP.

f Based on six cultures that met criteria for full identification and susceptibility testing.

g Culture reported as “*Staphylococcus* spp.” by BC-GP.

## Discussion

Diagnostic methods capable of reducing the time to identification and providing antimicrobial susceptibility results for agents of BSI have great potential to positively impact patient care. Molecular and fluorescent *in situ* hybridization (FISH) methods have been used to dramatically reduce the TAT for identification of a variety of organisms directly from positive blood cultures [15]–[18],[30]. Specifically, FISH has been used to identify and differentiate *S. aureus* from CoNS with a sensitivity of 96% to 98% and specificity of 89% to 100% within 30 min of culture positivity [15],[16]. FISH technology has also been used to identify *Enterococcus* spp. and various yeasts directly from positive blood cultures [31],[32]. Molecular methods for the detection of various bacteria including *P. aeruginosa*, *S. aureus*, and MRSA directly from blood culture have also been reported and demonstrate high sensitivity and specificity characteristics [17]–[19]. The use of currently available FISH and molecular methods are somewhat limited by the ability to identify only single or few specific targets (e.g., *Staphylococcus* spp., *Enterococcus* spp.). In this study, *Staphylococcus* spp. and *Enterococcus* spp. comprised 79.6% of bacteria isolated from positive blood cultures. Mass spectrometry based methods such as MALDI-ToF MS have the advantage of being able to identify nearly any bacterium or yeast present in a blood culture broth within approximately 30 min [12]–[14],[33]. A current weakness of this method is a limited ability to identify the individual constituents present in polymicrobial cultures [12]. Additionally, there are no widely available methods using MALDI-ToF to reliably identify genetic determinants of antimicrobial resistance, which is critical to selection of appropriate therapy. However, novel methods are currently in development that may enable phenotypic susceptibility testing for some organisms and antimicrobials [34],[35].

A multiplex, microarray-based assay (Prove-it sepsis assay, Mobidiag) has recently been described that is capable of identifying 50 bacterial pathogens and the *mecA* gene directly from positive blood culture broths with >94% sensitivity and >98% specificity [36]. Using this assay, authors were able to reduce TAT by 17.5 h to 43.1 h for the identification of bacteria in positive blood cultures as compared to routine culture methods. As with our study, accurate identification of all components in polymicrobial culture was challenging, suggesting a potential weakness for all multiplexed direct-from-specimen nucleic acid tests.

The BC-GP test is a sample to result microarray-based test consisting of single use reagents marketed at approximately US$75 USD per test. A major advantage of the BC-GP test is the rapid turnaround time following blood culture positivity. The BC-GP test requires only 350 µl of blood culture broth and can be completed within 2 h with less than 5 min of hands-on time. This enables same day analysis and reporting of positive blood culture results to physicians. The initial call rate including all prospective and contrived samples was 1,563/1,639 (95.4%). The BC-GP test panel includes 12 bacterial genus and species genetic targets representing the most common causes of Gram positive BSI. Only 87 monomicrobial cultures (7.5%) contained Gram positive organisms not present on the BC-GP test panel. This illustrates the broad applicability of this test for any blood culture containing Gram positive organisms upon primary stain. The most frequently isolated organisms not present on the BC-GP test panel were *Micrococcus* spp., *Corynebacterium* spp., *Kocuria* spp., *Bacillus* spp., and *Rothia* spp., which are common components of normal skin flora and are often associated with contaminated blood cultures [37].

*Staphylococcus* spp. are the most common species isolated from blood culture [38]. A recent study found that up to 32% of Staphylococcal BSIs were treated sub optimally using empiric therapy [39]. Identification and differentiation of MRSA, MSSA, and CoNS in positive blood cultures significantly reduces health care cost, shortens time to appropriate antimicrobial therapy, and improves patient outcome [10],[40]. A major benefit of the BC-GP test is the ability to identify and differentiate several species of *Staphylococcus*, including *S. aureus*, *S. epidermidis*, and *S. lugdunensis*, as well as the *mecA* gene encoding resistance to methicillin. Detection of MRSA using individual probes specific for *S. aureus* and *mecA* nucleic acid targets reduces the probability of false negative results that can occur as the result of genetic SCCmec rearrangements or emergence of new SCCmec cassettes [41],[42]. The ability to rapidly identify *S. lugdunensis* may also aid in guiding early patient management decisions because of the increased association of this bacterium with endocarditis, joint infections, and abscess compared to other CoNS [43],[44]. Additionally, accurate identification of *S. lugdunensis* is important to selection of antimicrobial therapy because of different breakpoints for oxacillin resistance in *S. lugdunensis* (≥4 µg/ml) compared to other CoNS (≥0.5 µg/ml) [25].

*Streptococcus* species are another group of organisms commonly isolated from blood cultures. Many alpha haemolytic “viridans group” species are relatively innocuous contaminants. In contrast, *S. pyogenes*, *S. agalactiae*, and *S. pneumoniae* can cause serious infection including meningitis, necrotizing fasciitis, and pneumonia. The BC-GP test misidentified five viridans group streptococci as *S. pneumoniae*. This is likely due to the close genetic relationship between mitis (viridans) group streptococci and *S. pneumoniae*. However, the overall specificity of the BC-GP test was still 99.6% for identification of *S. pneumoniae* in blood culture broth. Blood cultures containing *S. pneumoniae* as identified by the BC-GP test may be confirmed using standard biochemical tests such as optochin disk or bile solubility test once the isolate is cultured on solid medium. Blood culture is not recommended for the routine diagnosis of community acquired pneumonia (CAP) [45]; however in patients presenting with severe CAP, 2.1% to 13% were also bacteremic [46],[47]. Culture-based identification of the infecting organism from patient blood samples resulted in modification of antimicrobial therapy in 67% to 83% of these cases, including de-escalation in 50% to 80% of cases [46],[47]. Use of a direct-from-blood culture broth identification method such as the BC-GP test could enable earlier therapy interventions in these cases. Invasive infections with *S. agalactiae* are becoming more prevalent in non-pregnant adults including the elderly and immunocompromised [48],[49]. In one study central nervous system (CNS) involvement was present in 15% of patients with positive blood cultures and in 54.5% of disseminated infections [48]. In another case series, 78% of patients with CNS disease also had blood cultures positive for *S. agalactiae* [50]. Early identification of these organisms directly from blood culture broth also enables targeted antimicrobial therapy since these species are almost universally susceptible to penicillin and cephalosporins.

*Enterococcus* species, specifically *E. faecium* and *E faecalis*, was the third most commonly isolated genus in this study and is the second leading cause of BSI [38]. *E. faecalis* is more commonly isolated in blood cultures and is typically more susceptible to antimicrobials than *E. faecium* [51]. Specifically, resistance to ampicillin and vancomycin are rare in *E. faecalis*, 1.3%, and 0.5%, respectively, while 82.4% and 9.6% of *E. faecium* isolates are resistant to ampicillin and vancomycin, respectively [51]. Use of vancomycin as empiric therapy for suspected BSI has led to VRE being the most frequent agents of BSI that are inadequately treated [38]. The ability to identify and differentiate *E. faecalis* and *E. faecium*, along with the *vanA* and *vanB* genes directly from positive blood culture broth has the potential to dramatically reduce the time to appropriate therapy for these organisms.

Polymicrobial blood cultures accounted for 7.6% of prospectively collected blood cultures in this study. Cultures containing multiple organisms can be a challenge for direct detection methodologies for several reasons. In the case of MALDI-ToF, cumulative protein profiles of multiple organisms must be deconvoluted before accurate comparison to single reference spectra can be made. For molecular tests that detect a limited number of targets only one of the constituents may be reported. This can be misleading in the case of cultures containing multiple organisms with similar Gram stain morphologies. The use of microarray-based detection by the BC-GP test allows the capture and detection of multiple analytes, which decreases the chance of missing individual organisms in a polymicrobial culture. Agreement between the BC-GP test and reference culture method for polymicrobial cultures was only 72%. This is substantially lower than the 98% agreement observed for bacterial targets in prospective monomicrobial cultures. The majority of discrepant results in polymicrobial cultures involved CoNS, including *S. epidermidis*, which were missed by the BC-GP test. The increased prevalence of false negative results in polymicrobial cultures could be due to technical limitations of the test such as signal interference between multiple capture probes on the array. False negative results could also be due to minor constituents of the polymicrobial culture being present in low quantity, near the limit of detection of the BC-GP test.

A retrospective chart review of 107 blood culture broths containing Gram-positive organisms analyzed by BC-GP test demonstrated the ability to differentiate *S. aureus* from common blood culture contaminants including CoNS and other organisms with similar Gram-stain morphologies (e.g., *Micrococcus* spp, *Rothia* spp.) an average of 27.8 h (range 16 to 48 h) earlier than differentiation using routine solid media culture-based methods. Further, the identification of MRSA and MSSA were made 31 to 68 h (average 42.2 h) earlier using the BC-GP test as compared to routine culture identification and antimicrobial susceptibility testing. These data are similar to results reported by Bauer et al. and Ly et al. [10],[52]. These authors demonstrated a US$19,000 to US$21,000 reduction in cost of care, 2- to 6-d reduction in ICU stay, and a reduction in sepsis related mortality from 16.8% (usual care) to 7.9% (early identification) using other direct-from-blood culture identification methods to identify and differentiate *Staphylococcus* spp., including MRSA and MSSA. An additional advantage of the BC-GP test is the ability to also identify *E. faecalis* and *E. faecium*, along with the *vanA* and *vanB* genes encoding vancomycin resistance. VRE are not covered by routinely used empiric therapy for BSI. Upon primary Gram stain, these resistant organisms appear very similar to *Streptococcus* spp., which are susceptible to vancomycin often included as part of routine empiric therapy. In this study, use of the BC-GP test was able to reduce the time to identification of VRE by 42 to 44 h. The rapid times to identification of MRSA, MSSA, and VRE enable earlier, targeted, modification to empiric therapy, which can result in improved patient outcome [10],[52].

One weakness of the BC-GP test is the inability to assign *mecA* positivity to a specific organism in a mixed culture. In the case of a culture positive for “*S. aureus*,” “*S. epidermidis*,” and “*mecA*,” the laboratory would have to wait for solid media-based isolation of each organism and susceptibility testing to determine which organism was methicillin resistant. This would delay antimicrobial de-escalation in the case of a culture containing MSSA that was contaminated with methicillin resistant *S. epidermidis*. This scenario appears to be rare however, occurring in only 1/1,252 (0.07%) prospectively collected cultures in this study. A second weakness of the BC-GP test is the omission of additional targets from the test panel that are common contaminants in blood cultures. Specifically, *Micrococcus spp*. was present in 26/87 (29.9%) of cultures containing organisms not on the BC-GP test panel. In addition to being a common contaminant, the Gram stain morphology of *Micrococcus* can be similar to that of *Staphylococcus.* This may cause hesitation in reporting cultures containing Gram positive cocci in clusters as “negative for Staphylococcus” even if all *Staphylococcus* targets are reported as “not detected” by BC-GP.

There were also two potential weaknesses in the design of this study that may have influenced sensitivity and specificity characteristics for specific targets. First, species level identification of CoNS was achieved using the Vitek 2 bacterial identification system. Because of similar biochemical profiles, the accuracy of Vitek 2 for identification of CoNS species ranges from 86% to 87.5% [53],[54]. The observed sensitivity of BC-GP to identify *S. epidermidis* was only 96.5%, which was lower than other *Staphylococcus* targets (99.4% to 100% sensitive). In 7/10 cultures containing *S. epidermidis* according to Vitek 2 result the BC-GP reported “*Staphylococcus* spp.” only, apparently missing the *S. epidermidis* target. These false negative results could be due to failure of the BC-GP test to accurately identify the *S. epidermidis*-specific target, but could also be the result of misidentification by Vitek 2. The second potential weakness is the use of cefoxitin disk diffusion method as a surrogate test to indicate the presence of *mecA* in *S. aureus* and *S. epidermidis*. False positive results (i.e., *mecA* positive/cefoxitin susceptible) have been attributed specific alleles of the *mecA* repressor, *mecI*, or point mutations in the *mecA* promoter region that decrease resistance to cefoxitin in strains of *S. aureus* carrying a functional *mecA* [55]; however, additional mechanisms may also contribute to this phenotype [56]. Conversely, false negative results, i.e., BC-GP test negative/cefoxitin resistant, could be due to resistance mechanisms other than *mecA* [57],[58]. Amplification and nucleic acid sequencing of the *mecA* gene would be required to definitively characterize these isolates. We addressed these weaknesses using nucleic acid sequencing for definitive identification of any discrepant result (false positive or false negative) involving *S. epidermidis* or the *mecA* gene.

The strengths of this study include the large enrollment of prospectively collected blood cultures (*n = *1,252) by five geographically distinct clinical centers. Four of five sites (sites A–D) used BACTEC Aerobic/F broth and one site (site E) used BacT/ALERT FA aerobic broth for blood culture prior to testing on BC-GP. The sensitivity and specificity for each BC-GP target at individual clinical sites was within the 95% confidence interval for all sites combined, indicating that the BC-GP test generates reliable results independent of external variables such as geographic region, blood culture media, test operator, and prevalence of specific agents of BSI. Each target on the BC-GP panel was tested a minimum of 33 times using unique blood cultures containing each of the targets. For rare targets such as *Listeria* and *vanB*, contrived cultures prepared using clinical isolates were utilized to fully vet the test performance. The *van* gene of all *Enterococcus* isolates demonstrating phenotypic resistance to vancomycin was sequenced to confirm the BG-GP result of *vanA* or *vanB* as well as to determine the specific genetic variant of the gene present in each isolate.

We report the performance of the FDA cleared BC-GP test for the identification of Gram-positive organisms directly from positive blood culture broths. The high sensitivity and specificity characteristics of this test, coupled with on-demand testing capability and a 2-h TAT enable reporting of both the identification and antimicrobial resistance genes of bacteria obtained from blood culture significantly faster than using routine culture methods.

## Supporting Information

Figure S1 **Flowchart for reference testing of isolates obtained from positive blood cultures.**
(TIF)

Table S1 **STARD checklist for reporting of studies of diagnostic accuracy.**
(DOC)

Table S2 **Sequence results for** ***vanA*** **in prospectively tested blood cultures containing** ***Enterococcus spp*** **.**
(DOCX)

Text S1 **Full study protocol including inclusion and exclusion criteria, BC-GP test method, and routine culture method used in this study.**
(DOCX)

## Abbreviations

BC-GP: Gram-Positive Blood Culture test
BSI: bloodstream infection
CoNS: coagulase negative *Staphylococcus* spp
ICU: intensive care unit
MALDI-ToF MS: matrix assisted laser desorption ionization time-of-flight mass spectrometry
MRSA: methicillin-resistant *S. aureus*
MSSA: methicillin susceptible *S. aureus*
TAT: turn-around-time
VRE: vancomycin-resistant *Enterococcus*
