# Supplementary figures and images for: Multiplex Identification of Gram-Positive Bacteria and Resistance Determinants Directly from Positive Blood Culture Broths: Evaluation of an Automated Microarray-Based Nucleic Acid Test
Source: PLoS Med. 2013 Jul 2;10(7):e1001478. doi: 10.1371/journal.pmed.1001478 (PMC3699453; doi:10.1371/journal.pmed.1001478)

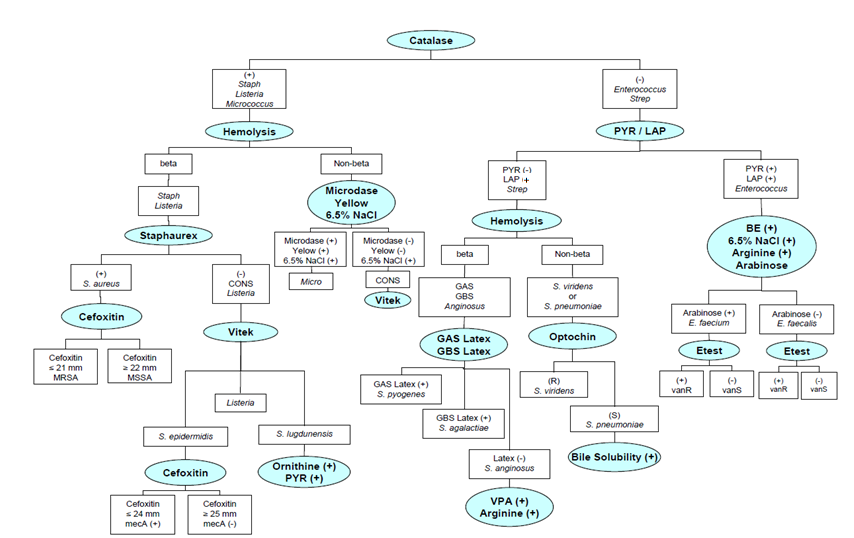

Supplement: Figure S1 — Flowchart for reference testing of isolates obtained from positive blood cultures. (TIF) [file pmed.1001478.s001.tif]
