# Supplementary material for: Multiplex Identification of Gram-Positive Bacteria and Resistance Determinants Directly from Positive Blood Culture Broths: Evaluation of an Automated Microarray-Based Nucleic Acid Test
Source: PLoS Med. 2013 Jul 2;10(7):e1001478. doi: 10.1371/journal.pmed.1001478 (PMC3699453; doi:10.1371/journal.pmed.1001478)
Supplement: Table S2 — Sequence results for vanA in prospectively tested blood cultures containing Enterococcus spp . (DOCX) [file pmed.1001478.s003.docx]

Table S2. Sequence results for prospectively tested *vanA* positive *Enterococcus spp*. isolates.

| **Site^a^** | **Sample ID** | **Reference Lab Final ID^b^** | **BC-GP   FInal Result^c^** | ***E. faecium*^d^** | ***E. faecalis*^d^** | ***vanA*^d^** | ***vanB*^d^** | **Bottle Type^e^** | **Sequencing Result** |
| --- | --- | --- | --- | --- | --- | --- | --- | --- | --- |
| MCW | A0012 | *E. faecium*(vanR) | Not Detected | FN | TN | FN | TN | BT A+ | vanA-Variant2 |
| MCW | A0015 | *E. faecalis*(vanS) | *E. faecalis* | TN | TP | TN | TN | BT A+ | N/A |
| MCW | A0035 | *E. faecalis*(vanS) | *E. faecalis* | TN | TP | TN | TN | BT A+ | N/A |
| MCW | A0037 | *E. faecalis*(vanS) | *E. faecalis* | TN | TP | TN | TN | BT A+ | N/A |
| MCW | A0058 | *E. faecalis*(vanS) | *E. faecalis* | TN | TP | TN | TN | BT A+ | N/A |
| MCW | A0086 | *E. faecalis*(vanS) | *E. faecalis* | TN | TP | TN | TN | BT A+ | N/A |
| MCW | A0089 | *E. faecalis*(vanS) | *E. faecalis* | TN | TP | TN | TN | BT A+ | N/A |
| MCW | A0090 | *E. faecalis*(vanS) | *E. faecalis* | TN | TP | TN | TN | BT A+ | N/A |
| MCW | A0098 | *E. faecalis*(vanS) | *E. faecalis* | TN | TP | TN | TN | BT A+ | N/A |
| MCW | A0117 | *E. faecalis* (vanS) | *E. faecalis* | TN | TP | TN | TN | BT A+ | N/A |
| MCW | A0141 | *E. faecalis*(vanS) | *E. faecalis* | TN | TP | TN | TN | BT A+ | N/A |
| MCW | A0014 | *E. faecium*(vanS) | *E. faecium* | TP | TN | TN | TN | BT A+ | N/A |
| MCW | A0046 | *E. faecium*(vanR) | *E. faecium & vanA* | TP | TN | TP | TN | BT A+ | vanA-Variant2 |
| MCW | A0062 | *E. faecium*(vanR) | *E. faecium & vanA* | TP | TN | TP | TN | BT A+ | vanA-Variant2 |
| MCW | A0138 | *E. faecium*(vanR) | *E. faecium & vanA* | TP | TN | TP | TN | BT A+ | vanA-Variant2 |
| MCW | A0139 | *E. faecium*(vanR) | *E. faecium & vanA* | TP | TN | TP | TN | BT A+ | vanA-Variant2 |
| OSU | B0121 | *E. faecalis* (vanS) | Not Detected | TN | FN | TN | TN | BT A+ | N/A |
| OSU | B0015 | *E. faecalis*(vanR) | *E. faecalis & vanA* | TN | TP | TP | TN | BT A+ | vanA-Variant2 |
| OSU | B0004 | *E. faecalis*(vanS) | *E. faecalis* | TN | TP | TN | TN | BT A+ | N/A |
| OSU | B0024 | *E. faecalis*(vanS) | *E. faecalis* | TN | TP | TN | TN | BT A+ | N/A |
| OSU | B0026 | *E. faecalis*(vanS) | *E. faecalis* | TN | TP | TN | TN | BT A+ | N/A |
| OSU | B0071 | *E. faecalis*(vanS) | *E. faecalis* | TN | TP | TN | TN | BT A+ | N/A |
| OSU | B0119 | *E. faecalis*(vanS) | *E. faecalis* | TN | TP | TN | TN | BT A+ | N/A |
| OSU | B0120 | *E. faecalis*(vanS) | *E. faecalis* | TN | TP | TN | TN | BT A+ | N/A |
| OSU | B0125 | *E. faecalis*(vanS) | *E. faecalis* | TN | TP | TN | TN | BT A+ | N/A |
| OSU | B0021 | *E. faecalis*(vanS) | *E. faecalis* | TN | TP | TN | TN | BT A+ | N/A |
| OSU | B0204 | *E. faecalis*(vanS) | *E. faecalis* | TN | TP | TN | TN | BT A+ | N/A |
| OSU | B0208 | *E. faecalis*(vanS) | *E. faecalis* | TN | TP | TN | TN | BT A+ | N/A |
| OSU | B0027 | *E. faecium*(vanR) | *E. faecium & vanA* | TP | TN | TP | TN | BT A+ | vanA-Variant2 |
| OSU | B0056 | *E. faecium*(vanR) | *E. faecium & vanA* | TP | TN | TP | TN | BT A+ | vanA-Variant2 |
| OSU | B0093 | *E. faecium*(vanR) | *E. faecium & vanA* | TP | TN | TP | TN | BT A+ | vanA-Variant2 |
| OSU | B0096 | *E. faecium*(vanR) | *E. faecium & vanA* | TP | TN | TP | TN | BT A+ | vanA-Variant2 |
| OSU | B0118 | *E. faecium*(vanR) | *E. faecium & vanA* | TP | TN | TP | TN | BT A+ | vanA-Variant2 |
| OSU | B0181 | *E. faecium*(vanR) | *E. faecium & vanA* | TP | TN | TP | TN | BT A+ | vanA-Variant2 |
| OSU | B0185 | *E. faecium*(vanR) | *E. faecium & vanA* | TP | TN | TP | TN | BT A+ | vanA-Variant2 |
| OSU | B0205 | *E. faecium*(vanR) | *E. faecium & vanA* | TP | TN | TP | TN | BT A+ | vanA-Variant2 |
| OSU | B0068 | *E. faecium*(vanS) | *E. faecium* | TP | TN | TN | TN | BT A+ | N/A |
| NSU | F0060 | *E. faecalis*(vanR) | Not Detected | TN | FN | FN | TN | BT A+ | vanA & vanB Sequencing negative |
| NSU | F0026 | *E. faecalis*(vanS) | *E. faecalis* | TN | TP | TN | TN | BT A+ | N/A |
| NSU | F0088 | *E. faecalis*(vanS) | *E. faecalis* | TN | TP | TN | TN | BT A+ | N/A |
| NSU | F0102 | *E. faecalis*(vanS) | *E. faecalis* | TN | TP | TN | TN | BT A+ | N/A |
| NSU | F0103 | *E. faecalis*(vanS) | *E. faecalis* | TN | TP | TN | TN | BT A+ | N/A |
| NSU | F0114 | *E. faecalis*(vanS) | *E. faecalis* | TN | TP | TN | TN | BT A+ | N/A |
| NSU | F0124 | *E. faecalis*(vanS) | *E. faecalis* | TN | TP | TN | TN | BT A+ | N/A |
| MN | E0302 | *E. faecalis* | *E. faecalis* | TN | TP | TN | TN | BTA FA | N/A |
| MN | E0053 | *E. faecalis* (vanS) | *E. faecalis* | TN | TP | TN | TN | BTA FA | N/A |
| MN | E0233 | *E. faecalis* (vanS) | *E. faecalis* | TN | TP | TN | TN | BTA FA | N/A |
| MN | E0011 | *E. faecalis*(vanS) | *E. faecalis* | TN | TP | TN | TN | BTA FA | N/A |
| MN | E0041 | *E. faecalis*(vanS) | *E. faecalis* | TN | TP | TN | TN | BTA FA | N/A |
| MN | E0228 | *E. faecalis*(vanS) | *E. faecalis* | TN | TP | TN | TN | BTA FA | N/A |
| MN | E0261 | *E. faecalis*(vanS) | *E. faecalis* | TN | TP | TN | TN | BTA FA | N/A |
| MN | E0309 | *E. faecalis*(vanS) | *E. faecalis* | TN | TP | TN | TN | BTA FA | N/A |
| MN | E0085 | *E. faecalis*(vanS) | *E. faecalis* | TN | TP | TN | TN | BTA FA | N/A |
| MN | E0285 | *E. faecalis*(vanS) | *E. faecalis* | TN | TP | TN | TN | BTA FA | N/A |
| MN | E0336 | *E. faecalis*(vanS) | *E. faecalis* | TN | TP | TN | TN | BTA FA | N/A |
| MN | E0124 | *E. faecium*(vanR) | *E. faecium & vanA* | TP | TN | TP | TN | BTA FA | vanA-Variant2 |
| MN | E0268 | *E. faecium*(vanR) | *E. faecium & vanA* | TP | TN | TP | TN | BTA FA | vanA-Variant2 |
| MN | E0293 | *E. faecium*(vanR) | *E. faecium & vanA* | TP | TN | TP | TN | BTA FA | vanA-Variant2 |
| MN | E0248 | *E.faecalis*(vanS) | *E. faecalis* | TN | TP | TN | TN | BTA FA | N/A |
| LIJ | D0027 | *E. faecalis* (vanS) | *E. faecalis* | TN | TP | TN | TN | BT A+ | N/A |
| LIJ | D0037 | *E. faecalis*(vanS) | *E. faecalis* | TN | FN | TN | TN | BT A+ | N/A |
| LIJ | D0114 | *E. faecalis*(vanS) | *E. faecalis* | TN | TP | TN | TN | BT A+ | N/A |
| LIJ | D0134 | *E. faecalis*(vanS) | *E. faecalis* | TN | TP | TN | TN | BT A+ | N/A |
| LIJ | D0151 | *E. faecalis*(vanS) | *E. faecalis* | TN | TP | TN | TN | BT A+ | N/A |
| LIJ | D0215 | *E. faecalis*(vanS) | *E. faecalis* | TN | TP | TN | TN | BT A+ | N/A |
| LIJ | D0218 | *E. faecalis*(vanS) | *E. faecalis* | TN | TP | TN | TN | BT A+ | N/A |
| LIJ | D0226 | *E. faecalis*(vanS) | *E. faecalis* | TN | TP | TN | TN | BT A+ | N/A |
| LIJ | D0289 | *E. faecium*(vanR) | Not Detected | FN | TN | FN | TN | BT A+ | vanA-Variant2 |
| LIJ | D0311 | *E. faecalis*(vanS) | *E. faecalis* | TN | TP | TN | TN | BT A+ | N/A |
| LIJ | D0312 | *E. faecalis*(vanS) | *E. faecalis* | TN | TP | TN | TN | BT A+ | N/A |
| LIJ | D0330 | *E. faecalis*(vanS) | *E. faecalis* | TN | TP | TN | TN | BT A+ | N/A |
| LIJ | D0334 | *E. faecalis*(vanS) | *E. faecalis* | TN | TP | TN | TN | BT A+ | N/A |
| LIJ | D0378 | *E. faecalis*(vanS) | *E. faecalis* | TN | TP | TN | TN | BT A+ | N/A |
| LIJ | D0390 | *E. faecalis*(vanS) | *E. faecalis* | TN | TP | TN | TN | BT A+ | N/A |
| LIJ | D0394 | *E. faecalis*(vanS) | *E. faecalis* | TN | TP | TN | TN | BT A+ | N/A |
| LIJ | D0233 | *E. faecalis*(vanR) | *E. faecalis & vanA* | TN | TP | TP | TN | BT A+ | vanA-Variant2 |
| LIJ | D0328 | *E. faecalis*(vanR) | *E. faecalis & vanA* | TN | TP | TP | TN | BT A+ | vanA-Variant2 |
| LIJ | D0321 | *E. faecium*(vanS) | *E. faecium* | TP | TN | TN | TN | BT A+ | N/A |
| LIJ | D0082 | *E. faecium*(vanR) | *E. faecium & vanA* | TP | TN | TP | TN | BT A+ | vanA-Variant2 |
| LIJ | D0159 | *E. faecium*(vanR) | *E. faecium & vanA* | TP | TN | TP | TN | BT A+ | vanA-Variant2 |
| LIJ | D0199 | *E. faecium* (vanR) | *E. faecium & vanA* | TP | TN | TP | TN | BT A+ | vanA-Variant2 |
| LIJ | D0204 | *E. faecium*(vanR) | *E. faecium & vanA* | TP | TN | TP | TN | BT A+ | vanA-Variant2 |
| LIJ | D0260 | *E. faecium*(vanR) | *E. faecium & vanA* | TP | TN | TP | TN | BT A+ | vanA-Variant2 |
| LIJ | D0267 | *E. faecium*(vanR) | *E. faecium & vanA* | TP | TN | TP | TN | BT A+ | vanA-Variant2 |
| LIJ | D0270 | *E. faecium*(vanR) | *E. faecium & vanA* | TP | TN | TP | TN | BT A+ | vanA-Variant2 |
| LIJ | D0309 | *E. faecium*(vanR) | *E. faecium & vanA* | TP | TN | TP | TN | BT A+ | vanA-Variant2 |

^a^Site at which sample was obtained and tested using the BC-GP test. MCW: Medical College of Wisconsin, OSU: Ohio State University, NSU: NorthShore University Health System, MN: medfusion, LIJ: North Shore-LIJ Health System

^b^Final identification and susceptibility based on reference culture results. VanS: vancomycin susceptible, VanR: vancomycin resistant

^c^Result reported by BC-GP test

^d^Interpretation of BC-GP test result based on reference culture method. TP: true positive, TN: true negative, FP: false positive, FN: false negative

^e^Blood culture bottle type. BT A+: BACTEC Plus Aerobic/F, BTA FA: BacT/ALERT FA FAN
