# Supplementary material for: Multiplex Identification of Gram-Positive Bacteria and Resistance Determinants Directly from Positive Blood Culture Broths: Evaluation of an Automated Microarray-Based Nucleic Acid Test
Source: PLoS Med. 2013 Jul 2;10(7):e1001478. doi: 10.1371/journal.pmed.1001478 (PMC3699453; doi:10.1371/journal.pmed.1001478)
Supplement: Text S1 — Full study protocol including inclusion and exclusion criteria, BC-GP test method, and routine culture method used in this study. (DOCX) [file pmed.1001478.s004.docx]

Text S1. Full study protocol

***1.0 Protocol Overview***

***1.1 Purpose***

This protocol guides the execution of a study utilizing the sample-to-result Verigene System and the Verigene Gram-Positive Blood Culture (BC-GP) Nucleic Acid Test.

The study will evaluate the analytical performance of the Verigene Gram-Positive Blood Culture (BC-GP) Nucleic Acid Test using the Verigene System, which is designed and manufactured by Nanosphere, Inc. of Northbrook, IL. The study will be conducted at five clinical centers.

***1.2 Test Overview***

The Verigene Gram-Positive Blood Culture (BC-GP) Nucleic Acid Test is a multiplexed assay that is designed to simultaneously detect and identify bloodstream infection (BSI) -causing, gram-positive bacteria. The test is performed on the Verigene System as a qualitative in vitro diagnostic test used for the identification of potentially pathogenic, gram-positive bacteria and is an aid to the identification of gram-positive bacteria bloodstream infections. It is not, however, used to monitor bloodstream infections.

The BC-GP test is performed directly on positive blood culture media specimens, using aerobic blood culture bottles, which are determined to have gram-positive bacterial growth by Gram stain.

The BC-GP test detects and identifies the following bacterial genera and species:

*·         Staphylococcus spp.*

*·         Streptococcus spp.*

*·         Micrococcus spp.*

*·         Listeria spp.*

*·         Staphylococcus aureus*

*·         Staphylococcus epidermidis*

*·         Staphylococcus lugdunensis*

*·         Streptococcus pneumonia*

*·         Streptococcus pyogenes*

*·         Streptococcus agalactiae*

*·         Streptococcus anginosus Group*

*·         Enterococcus faecalis*

*·         Enterococcus faecium*

In addition, the BC-GP test detects the *mecA* resistance marker, if present, in *Staphylococcus aureus*-positive specimens and *Staphylococcus epidermidis*-positive specimens.  The BC-GP test also detects the *vanA* or *vanB* resistance markers, if present, in *Enterococcus faecalis*-positive specimens and *Enterococcus faecium*-positive specimens.

***2.0 Verigene System Overview***

***2.1 Instruments***

The Verigene System is comprised of the Verigene Reader and the Processor SP.

The **Verigene Reader** is a bench-top, free-standing instrument with a touch screen control panel and a barcode scanner. It utilizes a graphical user interface to guide the user through the process of ordering tests and reporting results. There are no serviceable parts and no user calibration is required. Interaction with the touch screen is minimized through the use of barcodes. This instrument also controls the information for the specimens being processed and serves as the reader of test results. The key functions of the Reader include: 1) Entry and tracking of specimen identification numbers via manual keyboard input or barcode scanner, 2) Test selection for each specimen, 3) Automated transfer of specimen processing instructions on an assay-specific basis to Processor SP unit(s); a single Reader unit can control up to thirty-two Processor SP units, 4) Automated imaging and analysis of Test Cartridges, 5) Results display, and 6) Results report generation.

The **Verigene Processor SP** is capable of specimen preparation and target analyte detection under direction of the Reader. The Processor SP allows specimen extraction in addition to target analyte detection. The hybridization module and functionality are the same on the Processor SP as on the cleared Processor. Added to the Processor SP is an Extraction Module to extract nucleic acids from clinical specimens. The Processor SP itself contains no fluids and no user calibration is required. Reagents are provided in the Extraction Tray, Utility Tray, and the Test Cartridge. The operator only needs to add the consumables and the clinical specimen. Once the specimen is loaded by the operator, all other pipetting steps are performed by an automated pipettor that transfers reagents between wells of the tray(s) and finally loads the specimen into the Test Cartridge for hybridization.

***2.2 Consumables***

The **Extraction Tray** is a single-use disposable that contains all the reagents necessary to perform nucleic acid extraction from gram-positive blood culture media.

The **Utility Tray** is a single-use disposable that contains all the enzymatic reagents necessary to perform enzymatic digestion before nucleic acid extraction from gram-positive blood culture media. This tray also contains the process control 1 (PC1) tube.

Each **Test Cartridge** is a self-contained, single-use, laboratory consumable that consists of two parts: 1) a Reagent Pack, which is an upper housing unit preloaded with the reagents necessary for running the desired test and 2) the Substrate Holder. The Reagent Pack creates an air-tight hybridization chamber surrounding the region of the substrate containing the target-specific capture array.

***3.0 BC-GP Method Overview***

***3.1 Verigene System Process***

DNA extraction is performed on the Processor SP via an automated pipettor that transfers reagents between reagent wells in the Extraction Tray and the Utility Tray with a single-use pipette tip. Gram-positive blood culture media is placed in the specimen well of the extraction tray by the operator. The remaining steps of the assay are performed by the Processor SP. The Processor SP lyses the bacterial cells and cleaves proteins to access the nucleic acids. The nucleic acids are then bound to magnetic microparticles (MMPs), also referred to as magnetic glass particles. The MMP-bound nucleic acids are washed to remove cellular debris and lysis buffer contaminants. The nucleic acids are eluted off the MMPs in the presence of elution buffer and increased temperature.

The Processor SP pipettor mixes a pre-determined volume of extracted DNA with appropriate panel-specific specimen buffer and transfers this solution to the Test Cartridge. All of the hybridization steps take place within the Test Cartridge. Microfluidic channels and pumps control the addition and removal of reagents from the hybridization chamber. The target detection process occurs with hybridization of the target analyte to a synthetic gene-specific oligonucleotide capture strand on the hybridization cartridge’s substrate. A synthetic mediator target specific oligonucleotide is included with the test-specific buffer to form a hybridization “sandwich” with the gene sequence of interest. Washing steps following the target hybridization remove the unbound DNA from the hybridization chamber. A probe, composed of a gold nanoparticle with covalently bound oligonucleotides complementary to a sequence on the intermediate oligonucleotide, is introduced after the target wash. After the probe hybridization is completed, a series of washing steps remove the unbound probe from the hybridization chamber. A two-part signal enhancement reagent is added to the hybridization chamber and reacts with the gold nanoparticle to amplify the signal for the Verigene Reader scanning and analysis. Once the test is completed, the Test Cartridge is removed from the Processor SP unit and the Reagent Pack is removed and discarded. The Substrate is then imaged and analyzed on the Reader.

The Verigene Reader illuminates the signal-enhanced nanoparticles specifically bound to the bacteria-specific captures on the array. A photosensor reads the relative brightness of each spot and the Verigene Reader outputs a result based on relative levels of brightness of the capture signals. The Verigene Reader outputs a normalized value for the signal difference that allows for easy interpretation based on the signals detected by the Verigene Reader’s photosensor.

The net signal is the absolute signal level with the local background subtracted. Quality control checks in the call algorithm require that all of the array spots of each type be similar in signal intensity and have similar variation.

A negative control sequence is included to detect if the effective hybridization T_m_ (melt temperature) of the hybridization chamber is too low. This negative control will detect if the hybridization temperature is incorrect due to an instrument malfunction. The Verigene Reader automatically checks the target oligonucleotide signal relative to the negative control oligonucleotide signal. If the target signal is not adequately higher than the negative control signal, a “no call” result will be observed.

***4.0 Experimental Design***

The Study will evaluate the performance of the BC-GP test in comparison to a comparator reference method performed at a central clinical microbiology service laboratory (traditional culture/biochemical identification/susceptibility as relevant for each panel member). Cefoxitin will be used as a surrogate for detecting methicillin/oxacillin resistance. Bi-directional sequencing, performed by a third-party sequencing vendor, will be used to identify *vanA* and *vanB*.

The study will be conducted at 5 clinical centers. Each site will be provided with operation manuals, standard operating protocols, and “hands-on” training to ensure competency in executing the BC-GP test.

***4.1 Equipment and Materials***

***4.1.1 Provided by Nanosphere***

- Verigene Instruments:
  - Verigene Reader (Nanosphere Item #10-0000-02)
  - Verigene Processors SP (Nanosphere Item #10-0000-07 or #10-0000-08)
- Postscript 2 compatible printer; parallel or Ethernet network connection
- Training Specimens
- Nuclease-free 2.0 mL cryovials and 0.6 mL microcentrifuge tubes
- BBL Prepared Tubed Media for Long-Term Maintenance of Microorganisms Trypticase Soy Broth with 20% Glycerol (BD#297808)
- Specimen, plate, and tube identification labels
- Sequencing services at a third site as needed (in particular to differentiate *vanA* and *vanB* isolates if vancomycin resistance is present)
- Culture identification services at a third site as needed
- Verigene BC-GP test supplies:
  - BC-GP (BSI+) Extraction Trays/Tip Holder Assemblies
  - BC-GP (BSI+) Test Cartridges
  - BC-GP (BSI+) Utility Trays
  - BC-GP (BSI+) Processing Control 1 Tubes

***4.1.2 Provided by the Site***

- Standard lab supplies and equipment: pipettes and aerosol-resistant pipette tips, gloves, etc.
- 4°C Refrigerator
- ≤ -70°C Freezer
- 35°C Incubator
- Automated blood culture monitoring system: BACTEC™ 9000 series, BACTEC™ FX series, or BacT/ALERT® 3D series instrument (or similar)
- Blood culture media: BACTEC™ Plus Aerobic/F (92) Bottle, BacT/ALERT® FA FAN® Aerobic bottle (or similar)
- Gram staining reagents
- TSA with 5% Sheep Blood Agar (BAP) Plates (BBL 221261 or similar)

***4.1.3 Storage and Handling***

- BC-GP (BSI+) Test Cartridges: 2-8°C
- BC-GP (BSI+) Extraction Trays: 2-8°C
- BC-GP (BSI+) Utility Trays: 2-8°C
- BC-GP (BSI+) Processing Control 1 Tubes: 4°C
- Tip Holders: room temperature or 2-8°C
- Gram (+) Blood Culture Media Specimens: 2-8°C, ≤ -70°C
  - - Universal precautions must be taken when handling whole blood specimens.
- Extracted DNA: ≤ -70°C
- Glycerol stocks: ≤ -70°C

***4.2 Equipment Installation***

Nanosphere will install and test instruments prior to the clinical evaluation*.*

***4.3 Power Failure***

The instruments can be left on for the duration of the evaluation. If a power failure occurs, the Verigene Reader’s clinical session can be resumed when power is restored. If the Verigene Processor SP loses power within a procedure, then the specimen and reagents from that procedure are to be discarded. A new aliquot of the gram-positive blood culture media will be loaded if sufficient volume is available.

***4.4 BC-GP Controls***

***4.4.1 Positive Controls***

*Bacillus subtilis* serves as a “processing control” and is referred to as Processing Control 1 (PC1). The process control is added to each sample prior to the sample extraction step. The BC-GP test algorithm requires that the process control be detected before decisions regarding the presence or absence of any other target on the panel can be determined. If the process control is not detected a no call result will be obtained and the test should be repeated.

External positive controls (labeled as positive controls) in the form of bacteria specific to the test panel will be supplied to each test site for quality control purposes. For the study, external positive controls will be run at the following times:

- Training of study personnel.
- For the Methods Comparison Study—Once per day of testing.
- Any other time the study site PI directs that QC is warranted during the study period.

***4.4.2 Negative Controls***

Several test-specific negative controls are immobilized on the Test Substrate. These are used to guide test decisions. For a successful test, signals at the negative control sites should be below an empirically determined threshold. If not, this automatically disqualifies the test and no further analysis is performed regarding the presence or absence of the targets in the panel.

External negative controls (labeled as negative controls) will be supplied to each test site for quality control purposes. These will serve as a negative sample matrix control that would be processed through all the steps of the Verigene Test. For the study, external negative controls will be run at the following times:

- Training of study personnel.
- For the Methods Comparison Study—Once per day of testing.
- Any other time the study site PI directs that QC is warranted during the study period.

***5.0 Methods Comparison Study***

***5.1 Specimen Requirements and Management***

For the Methods Comparison Study, 100-300 deidentified specimens will be tested on the BC-GP test at each site.

The test panel contains analytes that vary widely in isolation frequency in a typical clinical microbiology laboratory. The isolation frequency also varies from laboratory-to-laboratory based on the patient population served and the blood culturing protocols utilized at the sites (number of sets of bottles to collect and how often to collect). Isolation frequencies of the organisms on the test panel range from higher frequency organisms (*S. aureus, S. epidermidis*) to very rare frequency organisms (*Listeria spp., Streptococcus pyogenes*).

Due to the various frequency rates of the test panel analytes the study sites will be testing a combination of specimens as follows: 1) Fresh prospective gram-positive blood culture media clinical specimens collected at each study site, 2) Frozen gram-positive blood culture media clinical specimens collected and shipped from a third-party specimen sourcing site, and 3) Frozen gram-positive blood culture media specimens prepared by the sponsor from glycerol stocks of clinical specimens.

Upon enrollment into the study, all specimens will be assigned a unique deidentification number for tracking purposes that will be utilized throughout the study. Fresh specimens will be collected at each study site guided by this protocol under IRB supervision. The specimen testing process for fresh specimens is listed below. Deidentified frozen specimens will be collected under IRB supervision at the specimen sourcing sites and shipped frozen to Nanosphere where they will be blinded and randomized prior to distribution to the study testing sites throughout the study. For very rare organisms, glycerol stocks will be obtained by Nanosphere, inoculated into blood culture media, grown to positivity, gram-stained, frozen, blinded and randomized to create contrived samples prior to distribution to the study testing sites throughout the study. The specimen testing process for all frozen specimens is listed below.

***5.2 Process for Testing Fresh Specimens (Refer to flow chart on Page 15):***

Each study site will de-identify the fresh propectively-collected specimen and assign a Nanosphere specimen ID number. Pre-printed labels will contain this de-identified Nanosphere specimen ID number and additional identifiers for labeling Tubes A-D as described below. Each site will be responsible for specimen blinding and de-identification of the fresh specimens that they are testing.

Gram Positive blood culture media will be aliquoted into Tubes A and B. Tube A (1.5mL of blood culture media) will be designated for Verigene Testing and Tube B (1mL of blood culture media) will be immediately stored frozen at ≤ -70°C.

To ensure homogeneity in the aliquots, the following process should be followed for each bottle type:

1). BACTEC™ Plus Aerobic/F (92) Bottles (or similar):

Invert the bottle several times to mix the media and insert the needle into the bottle using aseptic practices. Place the beveled edge of the needle against the sidewall of the bottles to prevent resin from being aspirated.

2). For BacT/ALERT® FA FAN® Aerobic bottle (or similar):

Invert the bottle several times to mix the media and insert the needle into the bottle using aseptic practices. The media will likely contain charcoal which is acceptable as long as the media is homogenous.

Each Verigene test requires 350uL of blood culture media and will take place within 12 hours of initial blood culture bottle positivity (as measured by the blood culture monitoring instrument).

Tube A will be stored at room temperature during Verigene testing. Tube A will be stored frozen at

≤ -70°C after repeat testing is completed (if needed). Repeat tests on the Verigene due to pre-analysis errors or no calls will be done using blood culture media from Tube A within 24 hours of initial blood culture bottle positivity. If the repeat test cannot be repeated within 24 hours, freeze Tube A at ≤ -70°C until testing can be done and follow the thawing directions in the frozen specimen processing section below.

Residual DNA from each Methods Comparison specimen test will be collected from the Verigene Extraction Tray and stored in Tube C at ≤ -70°C.

In addition, during the aliquoting process, a TSA with 5% Sheep Blood Agar Plate (BBL 221261 or equivalent) will be inoculated from the Gram-positive blood culture bottle. Following incubation at 34-36°C (35°C preferred) for 24 hours, two glycerol stocks will be made from each unique colony type on the plate. The plate will be reincubated for an additional 24 hours and observed for additional unique colony types. If additional unique colony types are observed then two glycerol stocks will be made. The glycerol stocks will be frozen at ≤ -70°C. One set of glycerol stocks will be retained at the study site and the second set of glycerol stocks will be shipped to a third-party service lab for confirmatory identification and methicillin/vancomycin susceptibility (if applicable) by traditional biochemical methods.

The third-party service lab will be responsible for plating the glycerol stocks, making a secondary set of glycerol stocks, and performing traditional biochemical analysis for confirmatory identification. Each unique colony type will also be tested on an automated identification card (e.g., VITEK, Phoenix or similar) to genus and species level by the third-party service laboratory. A methicillin AST or cefoxitin disk test (depending on common practices at the study site) will be performed on Staphylococcus aureus and Staphylococcus epidermidis isolates to determine methicillin resistance. A vancomycin AST or e-test (depending on common practices at the study site) will be performed on Entercoccus faecalis and Enterococcus faecium isolates to determine vancomycin resistance. The residual DNA from Tube C will be tested by bi-directional sequencing at a third-party sequencing laboratory for these vancomycin-resistant isolates to determine if vanA or vanB are present.

The table below provides a list of each tube (A-D), its designated contents, and use.

| Tube A | 1.5mL | Verigene Testing (350uL per test) |
| --- | --- | --- |
| Tube B | 1mL | Downstream Discrepancy Analysis (if required) |
| Tube C | - | Residual DNA from Verigene Extraction Tray |
| Tube D  Set 1 | - | Glycerol Stock retained at site. |
| Tube D  Set 2 | - | Glycerol Stock shipped to third-party service lab. |

All blood culture media specimens and DNA samples will be shipped to the sponsor on a pre-determined schedule under appropriate shipping conditions. All glycerol stocks will be shipped to the third-party service lab (or alternate site if necessary) on a pre-determined schedule under appropriate shipping conditions.

***5.3 Process for Testing Frozen Specimens (Refer to flow chart on Page 16):***

Frozen specimens will already have an assigned de-identified Nanosphere specimen ID number and will be labeled as Tube A. Pre-printed labels will contain this de-identified Nanosphere specimen ID number and additional identifiers for labeling Tubes C and D (the Tube B frozen aliquot will be stored at a separate site by the study sponsor) as described below.

Frozen gram positive blood culture media specimens will be thawed at room temperature (approximately 30 minutes) and inverted several times to mix (do not vortex). Ensure each aliquot is completely thawed before testing. Each Verigene test requires 350uL of blood culture media and will take place within 2 hours of thawing specimen.

Tube A will be stored at 4°C during Verigene testing. Tube A will be stored frozen at

≤ -70°C after repeat testing is completed (if needed). Repeat tests on the Verigene due to pre-analysis errors or no calls will be done using blood culture media from Tube A within 24 hours of thawing.

Residual DNA from each Methods Comparison specimen test will be collected from the Verigene Extraction Tray and stored in Tube C at ≤ -70°C.

In addition, during the aliquoting process, a TSA with 5% Sheep Blood Agar Plate (BBL 221261 or equivalent) will be inoculated from the thawed aliquot. Following incubation at 34-36°C (35°C preferred) for 24 hours, two glycerol stocks will be made from each unique colony type on the plate. The plate will be reincubated for an additional 24 hours and two glycerol stocks will be made from each additional unique colony type. The glycerol stocks will be frozen at ≤ -70°C. One set of glycerol stocks will be retained at the study site and the second set of glycerol stocks will be shipped to a third-party service lab for confirmatory identification and methicillin/vancomycin susceptibility (if applicable) by traditional biochemical methods. For plates with more than one unique colony type, a picture for documentation purposes will be obtained (camera to be provided by sponsor). If a picture cannot be obtained, a note stating why not is sufficient.

Each unique colony type will also be tested on an automated identification card (e.g. VITEK, Phoenix or similar) to genus and species level by the third-part service laboratory. A methicillin AST or cefoxitin disk test (depending on common practices at the study site) will be performed on Staphylococcus aureus and Staphylococcus epidermidis isolates to determine methicillin resistance. A vancomycin AST or e-test (depending on common practices at the study site) will be performed on Entercoccus faecalis and Enterococcus faecium isolates to determine vancomycin resistance. The residual DNA from Tube C will be tested by bi-directional sequencing at a third-party sequencing laboratory for these vancomycin-resistant isolates to determine if vanA or vanB are present.

The third-party service lab will be responsible for plating the glycerol stocks, making a secondary set of glycerol stocks, and performing traditional biochemical analysis for confirmatory identification.

The table below provides a list of each tube (A-D) and its designated contents and use.

| Tube A | 1.5mL | Verigene Testing (350uL per test) |
| --- | --- | --- |
| Tube C | - | Residual DNA from Verigene Extraction Tray |
| Tube D  Set 1 | - | Glycerol Stock retained at site. |
| Tube D  Set 2 | - | Glycerol Stock shipped to third-party service lab. |

All blood culture media specimens and DNA samples will be shipped to the sponsor on a pre-determined schedule under appropriate shipping conditions. All glycerol stocks will be shipped to the third-party service lab (or alternate site if necessary) on a pre-determined schedule under appropriate shipping condition.

***5.3 Inclusion/Exclusion Criteria***

***5.3.1 Inclusion Criteria***

- Specimens will either consist of prospectively collected fresh gram-positive blood culture media (BACTEC Plus Aerobic/F or BacTALERT Standard Aerobic blood culture bottles) that were collected following manufacturer’s package insert or prospectively collected retrospectively tested frozen gram-positive blood culture media.
- Specimens will be residual and de-identified.
- Fresh prospectively collected specimens will be tested within 12 hours of initial blood culture bottle positivity. In the case of a no-call or pre-analysis error, the specimen will be retested within 24 hours of initial blood culture positivity. If the specimen cannot be repeated within 24 hours if initial blood culture positivity, it should be frozen at ≤ -70°C until testing can be performed.
- Efforts to avoid multiple positive blood culture bottles from the same patient “septic episode” will be made, however, it is recognized that due to the de-identification process that is being utilized, several positive blood culture bottles from the same patient may be enrolled in the study.

***5.3.2 Exclusion Criteria***

- Inability to perform the reference or Verigene BC-GP Test on a specimen due to an analytical protocol violation (e.g., too little specimen, incorrect handling, use of incorrect blood culture bottle, no growth from glycerol stock at third-party service lab).
- Pediatric bottles will not be tested.
- Specimens collected off-label from the manufacturer’s package insert will be excluded (e.g., less than the minimum required blood volume was collected).

***5.4 Repeat Testing***

**5.4.1** If an error occurs during test processing that prevents a BC-GP Test substrate from being analyzed in the Verigene Reader, it is classified as a pre-analysis error. The recommended recourse for a pre-analysis error is one-time repeat testing of the specimen. Pre-analysis errors are to be excluded from call rate calculations but included in a pre-analysis error rate calculation.

**5.4.2** If a test results in a “no call”, the test should be repeated once as per the package insert and this result will be considered to be the final result for that specimen.

**5.4.3** If no residual DNA is present in the extraction tray after testing, the test should be repeated once.

**5.4.4** If a test results in a “no call-no grid”, verify the silver tape is removed from the slide and check that the orientation of the slide is flush with the holder. The slide may be re-scanned. If it cannot be rescanned the test should be repeated once.

***6.0 Data Analysis and Acceptance Criteria***

***6.1 Data Analysis***

At the end of the Methods Comparison study, the results will be reported in tabular form comparing the results from the BC-GP test to the Reference Method (culture and traditional biochemical method identification and susceptibility). Data analysis will consist of determining sensitivity and specificity with the reference traditional biochemical methods for each analyte tested on the BC-GP test. Two-sided 95% confidence intervals will be calculated for each analyte comparison.

All organisms will be directly identified from the gram-positive blood culture media by the Verigene BC-GP test at each testing site. The traditional biochemical reference methods that will be used for each analyte is listed in Attachment F and will be performed by the third-party service laboratory. In addition, every unique organism isolated on the BAP will also be identified to the genus/species level by an automated identification phenotypic system (Vitek) by the third-party service laboratory. These non-reference results will be utilized for discordant resolution.

The study will aim to test sufficient fresh and frozen (for the rarely isolated organism members of the panel) specimens representing each analyte included in the test panel such that they individually meet the sensitivity [i.e., 95% sensitivity with a lower bound of the two-sided 95% CI greater than 90% (92.5% for Staphylococcus aureus)] and specificity (i.e., 95% specificity with a lower bound of the two-sided 95% CI greater than 90%) acceptance criteria. The total sample size needed for the study will be estimated taking into account the frequency of isolation of each organism and these acceptance criteria.

***6.2 Discordant Results***

Discordant false positive and false negative results will be compared with results obtained on the automated identification phenotyping system (Vitek) at the third-party service laboratory. If a discordant result is not resolved by comparison with the phenotyping system result, then Tube B will be thawed, cultured, and subsequent identification of the specimen will be performed at the third-party service laboratory. Additionally, validated bi-directional sequencing methods may be used for discordant resolution.

***7.0 Data Recording***

All data and information are to be recorded concurrently with the data creation. No data is to be recorded from memory or transcribed from notes.

All data entries are to be clear, permanent, legible, accurate, complete and truthful.

All entries must be made directly onto the attached data sheet templates and must be initialed and dated.

Results from routine laboratory testing are to be completed by the clinical personnel. The required data to be entered are:

1. Specimen ID

2. Time and Date the blood culture bottle became positive

3. Time and Date the positive blood culture bottle was removed from the incubator

4. Time, Date, and Result of Gram staining

5. Type of Hemolysis observed in the Blood Agar Plate (BAP)

6. Number of glycerol stocks made from each specimen

***8.0 References***

**8.1** Verigene Gram-Positive Blood Culture (BC-GP) Nucleic Acid Test Investigational Use Only (IUO) Package Insert

**8.2** Verigene System User’s Manual (027-00002)

**8.3** BBL Prepared Tubed Media for Long-Term Maintenance of Microorganisms Trypticase Soy Broth with 20% Glycerol (BD#297808) Package Insert (8806331JAA)
